# Asymmetrical Two-Headed Linear Cationic Surfactants with Halogenoferrate Magnetic and Bromide Counterions: Synthesis, Thermal Behavior, Magnetic Performance, and Surface Properties

**DOI:** 10.1021/acs.langmuir.6c00730

**Published:** 2026-06-09

**Authors:** Łukasz Lamch, Maria Korabik, Dawid Szarpak, Przemysław Szklarz, Mariusz Borkowski, Piotr Warszyński, Kazimiera A. Wilk

**Affiliations:** † Faculty of Chemistry, 49567 Wrocław University of Science and Technology, Wybrzeże Wyspiańskiego 27, 50-370 Wrocław, Poland; ‡ Faculty of Chemistry, 49572 University of Wrocław, F. Joliot-Curie 14, 50-383 Wrocław, Poland; § Jerzy Haber Institute of Catalysis and Surface Chemistry, 49559 Polish Academy of Sciences, Niezapominajek 8, 30-239 Kraków, Poland

## Abstract

A series of multicharged surfactants with magnetic counterions (Mag-D-Surfs) containing one hydrophobic tail and two hydrophilic groupings, and comprising exclusively carbon atoms in the hydrophobic part, i.e., 2-alkyl-*N*,*N*,*N*,*N*′,*N*′,*N*′-hexamethylpropan-1,3-ammonium salts (ferrates; alkyl: decyl, dodecyl, and tetradecyl, abbreviated, respectively, as C_10_-D_C_Me_3_Mag, C_12_-D_C_NMe_3_Mag, and C_14_-D_C_NMe_3_Mag), were first synthesized and characterized by FT-IR, Raman, X-ray fluorescence (XRF), and FIR spectra as well as elemental analyses. Thermogravimetric analysis (TGA) and differential scanning calorimetry (DSC), supported by optical polarization microscopy, showed that the coexistence of isotropic and anisotropic subphases depended on cooling rates. Surface activity of C_*n*_-D_C_NMe_3_Mag at the air/water interface was evaluated by measuring the surface tension of their solutions by the pendant drop technique and compared with surfactants without magnetic function. Moreover, surface tensiometry in a magnetic field demonstrated that the studied Mag-D-Surfs exhibited magnetically induced changes in the drop shape. Their magnetic behavior in the solid state was determined by superconducting quantum interference device magnetometry (SQUID). All findings related to the aforementioned double-headed Mag-D-Surfs were compared to linear magnetic ionic liquids surfactants (MILSs), i.e., alkyltrimethylammonium halogenoferrates (alkyl: dodecyl [DTA]­[FeCl_*x*_Br_4–*x*_^–^], tetradecyl [TTA]­[FeCl_*x*_Br_4–*x*_^–^], and cetyl [CTA]­[FeCl_*x*_Br_4–*x*_^–^]). Magnetic tests confirm the paramagnetic nature of the studied compounds and the 1:1 molar ratio (surface active cation:Fe^3+^) for all the studied surfactants. Their unique physicochemical properties demonstrate exceptional performance, particularly in the development of new stimuli-responsive materials.

## Graphical abstract

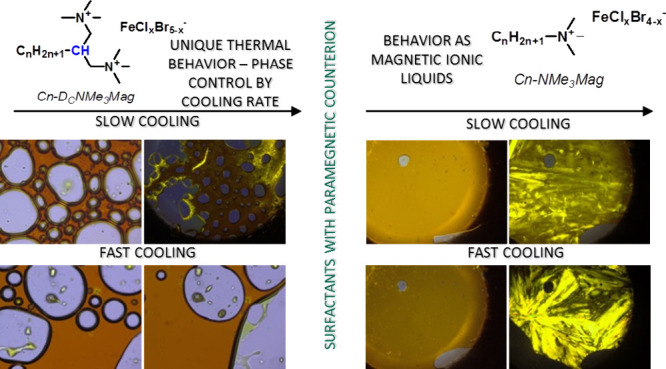

## Introduction

Molecular design of surfactants with widely varying architectures offers an excellent opportunity to tailor surfactant aggregation behavior and other physicochemical properties of their solutions, thereby enabling unique performance in industrial and academic fields. A majority of new multifunctional surfactants focused on fine and specialty chemicals are based on the premise that their unique relationships—chemical structure–self-organization properties at interfaces—lead to a variety of valuable new product functionalities, e.g., surface-active polyelectrolyte–surfactant complexes as new drug nanocarriers, surfactants with magnetic property, biologically active capped-metal nanoparticles, catalytic surfactants, corrosion inhibitors, or biocidal agents.^1–5^ Thus, various stimuli-responsive surfactants have been synthesized by introducing functional groups into their structures, which are sensitive to changes in pH, CO_2_ levels, light, and external field or to added enzymes or chemically labile systems that undergo bond cleavage.^6,7^ Among them, there are magnetic surfactants (MagSurfs) with magnetoresponsive function (coming from a high-spin transition metal or lanthanide cations), whose magnetic part can comprise the surfactant’s counterion or a fragment of the surfactant’s structure.^8–11^ In addition to the general single-head single-tail surfactants with one magnetic counterion, there are also described dimeric (so-called gemini) magnetic surfactants having two functional counterions in their structure as well as double-tailed or triple-tailed surfactants comprising two (or three) hydrophobic groupings and bivalent (or trivalent) countercations.^12–16^ MagSurfs possess lower values of critical micelle concentration (CMC) and better effectiveness in reducing surface tension as compared to their parent surfactants. The aqueous solutions of MagSurfs with sufficiently high concentration are paramagnetic, acquiring their magnetic behavior in the presence of a magnetic field. MagSurfs are superior to magnetic nanoparticles (MNPs) and their nonmagnetic analogues in numerous fields.^9,14^ In relation to MNPs, the following features can be recalled: favorable conditions for one-step synthesis, good stability and dispersibility in water, appropriate biocompatibility, and increased effective binding. Their potential to control non-invasively and reversibly their physicochemical properties by applying the external magnetic field gives remarkable opportunities for a variety of applications, including biotechnology, agriculture, drug delivery, catalysis, and water treatment, as well as various oil-derived industrial sectors for magnetic-driven delivery, adsorption, or separation.^9,17–22^ It should be emphasized that MagSurfs comprise a broad group of valuable chemicals: surfactants of natural origin with paramagnetic metal,^23^ stimuli-responsive compounds with controlled sol–gel transition,^24^ and unsaturated derivatives for enhanced oil recovery.^25^

It should be emphasized that for standard surfactants, their unique properties are exhibited in a dissolved state and, especially, at solid–liquid, liquid–liquid, or liquid–gas interfaces, due to their amphiphilic structure. A major drawback of studying surfactants in their bulky liquid or amorphous phases is their limited thermal stability, particularly for cationic and anionic salts.^14,26^ Simple quaternary ammonium salt-type surfactants, i.e., compounds with counterions of exclusively noncomplex origin like bromide or chloride, display several phase transitions at ambient temperatures (up to around 100–150 °C), although it is often difficult to study their behavior above ca. 175 °C due to their significant and rapid decomposition.^14,26,27^ For most common compounds, e.g., alkyltrimethylammonium bromides, the latter feature constitutes a lack of possibility for their complete liquefaction, followed by recrystallization from the bulk liquid phase. Introduction of the halogenoferrate counterion significantly lowers the melting point and improves thermal stability. It should be noted that it is unique for Fe. Introduction of any other complex counterion, e.g., cerium or gadolinium, does not provide melting point depression when compared with the parent cationic surfactant.^14^ Therefore, it is beneficial to introduce a halogenoferrate counterion into a cationic surfactant in order to gain unique thermal behavior with enhanced stability and magnetic function. Moreover, numerous quaternary ammonium salts with both simple or complex (e.g., halogenogadolinate or halogenocerate) counterions are characterized by relatively high Krafft points, limiting their performance in aqueous systems. Conversely, introduction of halogenoferrate counterion into quaternary ammonium group bearing surfactant molecule even improves solubility in water.

In this work, we present new perspectives on controlling the properties of halogenoferrate-borne surfactants by synthesizing a new group of multicharged structures (on the basis of dicephalic-type cationic surfactants with selectively introduced transition metal complexes, abbreviated as Mag-D-Surfs), containing one hydrophobic tail and two hydrophilic groupings as well as two appropriate (simple or complex) counterions in their structure and exclusively carbon atoms in the hydrophobic part (for their structures and abbreviations, see Table 1). Such Mag-D-Surfs would constitute a new class of specialty surfactants that have not yet been described in the literature with unique properties and performance, predominantly exhibited in the melted and the solidified state. Therefore, a group of multicharged Fe-derived magnetic surfactants were synthesized by combining parent dicephalic-type quaternary ammonium cationic surfactants (for the latter synthesis, see ref^28^) with Fe trichloride as the precursor of paramagnetic counterion. It should be emphasized that the synthetic procedures, including purification steps, were designed to assess unique phase behavior at elevated temperatures for Fe-containing surfactants, particularly in the context of potentially the highest crystalline subphase content. Since novel Mag-D-Surfs are particularly interesting as melts and solids, our experiments were designed to elucidate their structural properties by various spectroscopic methodologies (including extensive infrared and Raman spectroscopy supported by mass spectrometry), as well as thermal and magnetic behavior, while (typically performed for surfactants) experiments in aqueous systems aimed to show that such compounds did not lose their surface activity and responsiveness to external magnetic field. It has to be recalled that the presence of multiple polar groups in the surfactant molecule, by increasing the interaction strength between the cationic head and water, has a significant influence on the aggregation properties of surfactants in their aqueous environment.^4,29–32^ Thermogravimetric analysis (TGA) and differential scanning calorimetry (DSC) studies, supported by polarization microscopy investigations, were conducted to evaluate changes in the physicochemical properties of Mag-D-Surfs at elevated temperatures, particularly for their potential application as phase-change memory materials. This unique property opens the possibility to design novel types of data carriers applying a stable–metastable balance of the material’s subphases. Surface tension determination would make it possible to describe the behavior of Mag-D-Surfs at the interfaces, while superconducting quantum interference device (SQUID) magnetometry—their magnetic property and the presence of iron atoms in complex counterions. It should be emphasized that, in contrast to typical surfactants exhibiting unique properties in the dissolved state, MagSurfs performance is particularly visible in the solid/melted state, thus influencing our selection of investigation methodologies. Additionally, linear magnetic ionic liquids surfactants (MILSs), i.e., alkyltrimethylammonium halogenoferrates (alkyl: dodecyl [DTA]­[FeCl_*x*_Br_4–*x*_^–^], *x* = 3.517; tetradecyl [TTA]­[FeCl_*x*_Br_4–*x*_^–^], *x* = 3.783; and cetyl [CTA]­[FeCl_*x*_Br_4–*x*_^–^], *x* = 4), prepared in the analogous manner, constituted the referencing system for the studied multicharged Mag-D-Surfs in all the performed experiments.

**Table 1:**
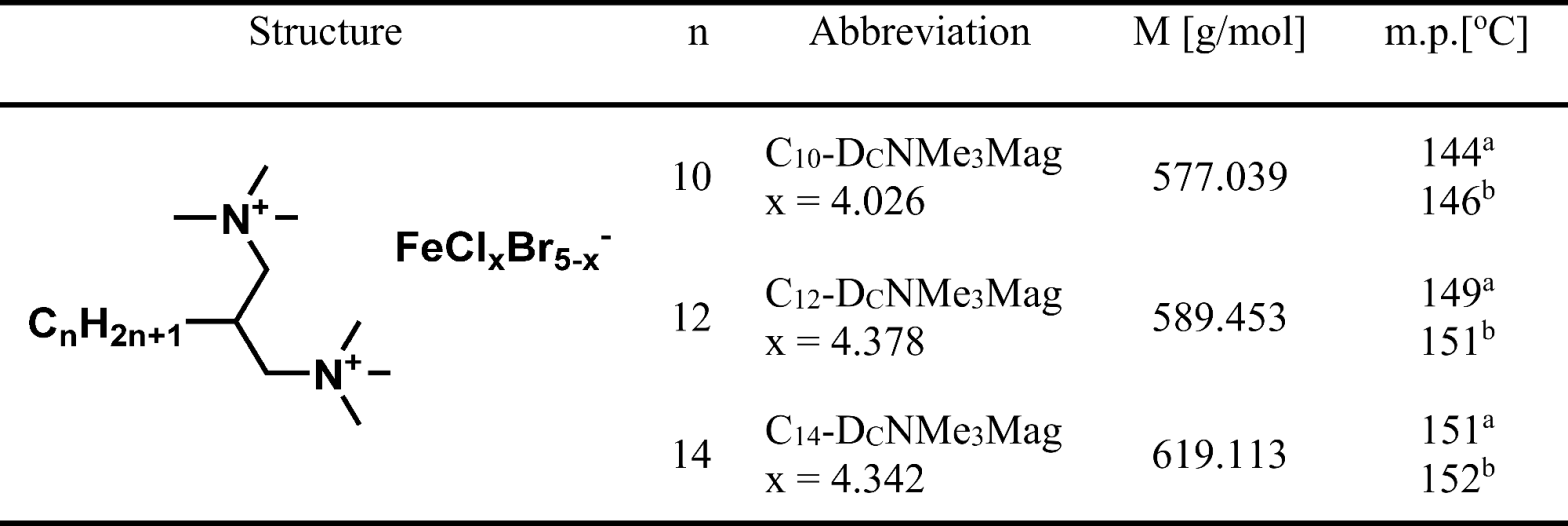
Designed Magnetic Dicephalic Surfactants (Mag-D-Surfs)—2-Alkyl-*N*,*N*,*N*,*N*′,*N*′,*N*′-hexamethylpropan-1,3-ammonium Halogenoferrates—Under Study

a Measured using the Boetius table (heating rate, 4 °C/min).

b Measured by DSC (endset; heating rate, 4 °C/min).

## Experimental Section

### Chemicals

All reagents used in the study were of at least analytical grade or better. Iron­(III) chloride hexahydrate was purchased from Sigma-Aldrich, USA. The commercially available alkyltrimethylammonium bromides were purchased from Sigma-Aldrich, USA (dodecyl, DTABr, purity > 98%) or from Carl Roth, Germany (tetradecyl, TTABr, purity > 6%; and hexadecyl, CTABr, purity > 99%). Magnetic ionic liquids surfactants (MILSs)—dodecyl-, tetradecyl-, and cetyltrimethylammonium halogenoferrates­(III) (i.e., [DTA]­[FeCl_*x*_Br_4–*x*_^–^], [TTA]­[FeCl_*x*_Br_4–*x*_^–^], and [CTA]­[FeCl_*x*_Br_4–*x*_^–^]—were synthesized as previously described^16,20^ and additionally purified by recrystallization at −20 °C from pure methanol ([CTA]­[FeCl_*x*_Br_4–*x*_^–^]) or a methanol–isopropanol mixture ([DTA]­[FeCl_*x*_Br_4–*x*_^–^] and [TTA]­[FeCl_*x*_Br_4–*x*_^–^]), followed by careful filtration and vacuum-drying. Chemical structures of intermediates and nonmagnetic parent surfactants were confirmed by ^1^H NMR spectroscopy. Water used in all experiments was double-distilled and purified using a Millipore (Bedford, MA) Milli-Q purification system.

### Synthesis of Double-Headed Magnetic Surfactants

Magnetic double-headed cationic surfactants (Mag-D-Surfs)—C_10_-D_C_NMe_3_Mag, C_12_-D_C_NMe_3_Mag, and C_14_-D_C_NMe_3_Mag—were synthesized from dicephalic cationic surfactant dibromides,^28^ i.e., 2-decyl-*N*,*N*,*N*,*N*′,*N*′,*N*′-hexamethylpropan-1,3-ammonium dibromide (abbreviated as C_10_-D_C_NMe_3_Br), 2-dodecyl-*N*,*N*,*N*,*N*′,*N*′,*N*′-hexamethylpropan-1,3-ammonium dibromide (C_12_-D_C_NMe_3_Br) and 2-tetradecyl-*N*,*N*,*N*,*N*′,*N*′,*N*′-hexamethylpropan-1,3-ammonium dibromide (C_14_-D_C_NMe_3_Br) by the reaction with iron­(III) chloride hexahydrate in methanolic solution. Briefly, appropriate dicephalic cationic dibromide (0.0123 mol) was dissolved in methanol (20 mL), followed by the gradual introduction of iron­(III) chloride methanolic solution (0.0250 mol in 15 mL of methanol) under intensive stirring. The reaction mixture was stirred for 24 h at room temperature in the dark. After the reaction was complete, an appropriate amount (ca. 45–55 mL) of isopropanol was introduced into the mixture, and the mixture was kept in a refrigerator (−20 °C) for 48–72 h until the precipitate formed. The obtained solid was carefully filtered, washed with three portions (3 × 10 mL) of cold isopropanol, and dried in vacuo. Yield: 55–65%. C_*n*_-D_C_NMe_3_Mag were purified by repeated recrystallization from a methanol–isopropanol mixture at reduced (−20 °C) temperature.

### Structure Characterization and Determination of Thermal Properties

#### Thermogravimetric Analysis (TGA)

Thermogravimetric analyses (TG-DSC) were performed using a Mettler-Toledo TGA/DSC 3+ thermogravimetric analyzer (Mettler-Toledo, Switzerland) operated under a nitrogen atmosphere at a heating rate of 4 °C/min in the range of 20–900 °C. The results are expressed as the temperature-dependent weight percent.

#### Differential Scanning Calorimetry (DSC)

DSC experiments were performed using a Mettler-Toledo DSC 3 (Mettler-Toledo, Switzerland) calorimeter equipped with a Mettler-Toledo liquid nitrogen cooling system, utilized to provide an inert gas atmosphere during the measurement in order to avoid any influence of oxygen on the sample behavior. Surfactants’ samples were loaded into aluminum pans and hermetically sealed, while empty pans were used as a reference. Samples were subjected to heating and cooling runs from 30 to 155 °C (Mag-D-surfs) or from −100 to 100 °C (MILSs) at varying rates of 4, 10, or 20 °C/min. Enthalpies of melting (Δ*H*_m_) and crystallization (Δ*H*_cr_), as well as the onset (denoted as crystallization temperature, *T*_cr_, for cooling curve), peak, and endset (denoted as melting temperature, *T*_m_, for heating curve) points were calculated utilizing STARe software (Mettler-Toledo). Heating–cooling cycles were designed in order to investigate possibility of crystalline/amorphous phase control by the appropriate thermal treatment of the sample.

#### Polarization Microscopy

Sample structures were assessed using the Olympus BX53 polarization microscope. The temperature change was introduced by a LINKAM THM-600 heating/cooling stage equipped with a heating/cooling table, enabling operation between −150 and 250 °C. Surfactants’ samples (around 10–15 mg) were heated up to their melting points (around 140 – 155 °C for Mag-D-Surfs or around 45–65 °C for MILSs) and cooled in a manner resembling DSC measurements (see subchapter Differential Scanning Calorimetry (DSC)) in order to determine purely amorphous or semicrystalline structure upon solidification.

#### Fourier Transform Infrared (FT-IR) Spectroscopy

Fourier transform infrared spectra were recorded in the 380–4000 cm^–1^ spectral range using an FT-IR spectrometer ALPHA II Bruker (Bruker Optics, Germany) equipped with a diamond ATR accessory.

#### Far Infrared (Far IR) Spectroscopy

Fourier transform infrared spectra were recorded in the 40–500 cm^–1^ spectral range using an FT-IR spectrometer Vertex 70 (Bruker Optics, Germany) as Nujol mulls in paraffin oil.

#### Elemental Analyses

Elemental analyses were performed utilizing the Vario EL cube (Elementar, Germany) calibrated with acetanilide, and utilizing samples’ masses between 2 and 7 mg. Each measurement was repeated at least in triplicate, and an average was taken as the result, with an absolute error not exceeding 0.3%. For the Cl assay, an ECD detector was used, and calibration was performed with benzylthiuronium chloride.

#### Raman Spectroscopy

Raman spectra in the 300–3200 cm^–1^ spectral range were assessed using a hand-held Raman spectrometer Bravo Bruker (Bruker Optics, Germany), enabling 785 and 852 nm laser excitation.

#### X-ray Fluorescence (XRF) Spectroscopy

X-ray fluorescence spectra were recorded on hand-held XRF spectrometers TRACER 5G Bruker (Bruker, Germany), equipped with a rhodium excitation source and SDD detector with a graphene window. The measurements were conducted with an acquisition time of 300 s, a voltage of 41 kV, and a current of 11 μA.

#### Electrospray Ionization Mass Spectrometry (ESI-MS)

Mass spectra were determined by utilizing electrospray ionization mass spectroscopy (ESI-MS) (micrOTOF-Q instrument; Bruker Daltonics, Germany). The ESI-MS apparatus was operated in positive-ion mode (calibrated with the Tunemix mixture; Bruker Daltonics; Germany). The spectra were analyzed by DataAnalysis 3.4 software (Bruker Daltonics, Germany) with a resolution of at least 5 ppm.

#### Nuclear Magnetic Resonance (NMR)

Proton nuclear magnetic resonance (^1^H NMR) spectra were determined on a Bruker AMX-500 spectrometer. Chemical shifts were given in ppm, while TMS (tetramethylsilane) was used as an internal standard. Compounds were dissolved in DMSO-*d*_6_ (all quaternary ammonium type surfactants) or CDCl_3_ (the obtained intermediates—exemplified for preparation of C_12_-D_C_NMe_3_Br) at concentrations 2–7 mg/mL. The recorded spectra were processed utilizing Bruker TopSpin software (version 3.6.1).

#### Surface Tension Measurements

The aqueous surfactant solutions were prepared immediately before measurement by diluting the stock solutions. Equilibrium surface tension measurements were performed using the pendant drop shape analysis method with the DSA25 expert goniometer (Krüss, Germany), equipped with a Peltier-controlled temperature chamber and humidity control, and the ADVANCE Software for data collection. Each solution, prepared around 1 h before studies, was measured at least in triplicate, and an average of the results with a relative error of less than 1% were taken for surface tension determination by fitting to the Young–Laplace equation. All surface tension measurements were performed at 295 K and a relative humidity of 75–90%. The effect of a magnetic field was investigated in a specially constructed setup in which a uniform magnetic field (up to 670 mT) was produced by water-cooled electromagnets and applied perpendicular to the pendant-drop axis. The changes in the drop shape were monitored by the camera in the direction perpendicular to the magnetic field. By fitting the Young–Laplace equation to the drop profile, the apparent changes in the surface tension were determined. For calculations, all magnetic surfactants were considered as pure compounds with the determined chemical composition. Such assumption was also confirmed by surface tension isotherms with no minima around CMC as well as all performed analyses, including FT-IR, FIR, Raman, and extensive TGA/DSC measurements.

#### Superconducting Quantum Interference Device (SQUID) Magnetometry

Measurements of magnetization were performed using an MPMS-3 Quantum Design SQUID magnetometer with an operating temperature equal to 300 K, with the values of the DC applied field ranging from −7 to 7 T. DC analysis was performed under a field of 0.5 T, using samples’ masses between 0.01562 and 0.04192 g. The effective magnetic moments were calculated from the expression and taken as an average of three different measurements

$$\mu_{\mathrm{eff}}=2.83\sqrt{\chi_{m}T}\left(B.M.\right)$$

where χ_m_ denotes mole magnetic susceptibility and *T*, absolute temperature.

## Results and Discussion

The recently synthesized and studied magnetic surfactants comprise three different types of structures: single-headgroup single-tail compounds with a magnetic counterion,^16,20^ gemini-type derivatives with doubled magnetic counterions of complex type,^9,14^ and multiheaded counterparts with chelated central metal ion.^19^ However, to the best of our knowledge, dicephalic-type surfactants have not yet been studied as magnetic group bearing amphiphiles, in contrast to gemini magnetic surfactants, which have been widely described.^1^ The design of our dicephalic magnetic surfactants is unique as they comprise exclusively carbon atoms in their structure, with the obvious exception of a quaternary nitrogen atom bearing hydrophilic headgroups and counterions. It is worth noticing that the utilized multistep synthetic route of modular origin involves the introduction of an appropriate chemical motif into a hydrophobic fragment, followed by its conversion into the one with the intended reactivity and, later, the final product. The modular approach enables further synthesis of surfactants with a multiplied number of headgroups by repeating the selected steps. The whole synthetic route of dicephalic magnetic surfactants (Mag-D-Surfs), with the marked final step—introduction of magnetic counterion—is shown in Scheme 1. Chemical structures of parent quaternary ammonium type surfactants of both linear (single-tail single-headgroup) or dicephalic architecture as well as intermediates (exemplified for the synthesis of C_12_-D_C_NMe_3_Br: C_12_-D_C_COOMe, C_12_-D_C_OH and C_12_-D_C_Br) were confirmed by ^1^H NMR spectroscopy and shown in Figures S1–S4.

**Scheme 1:**
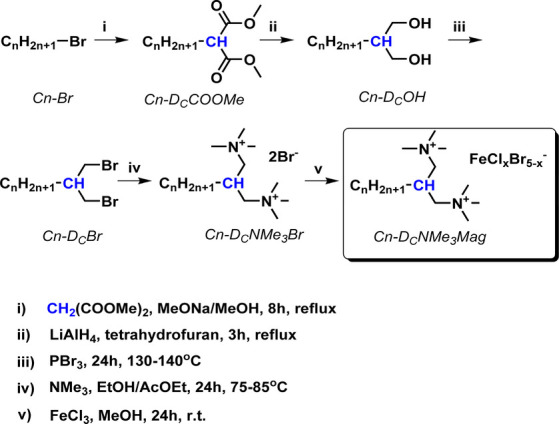
Synthesis of Dicephalic Magnetic Surfactants C_n_-D_C_NMe_3_Mag (*n* = 10, 12, 14)

The performed synthesis enabled obtaining Mag-D-Surfs in a convenient way, without the necessity to use harsh conditions for either preparation or purification. In contrast to standard procedures for isolating tetrahalogenoferrate-based magnetic surfactants, comprising evaporation of solvents to dryness,^12,15,16^ we introduced an appropriate crystallization step, utilizing a methanol–isopropanol mixture at −20 °C. Direct evaporation and drying yielded surfactants as viscous liquids, whereas crystallization enabled the formation of solid compounds. On the other hand, easy exchange of Br and Cl in complex counterions led to the formation of solid compounds characterized by varying bromine-to-chlorine ratios. Standard MILSs crystallized as compounds with a positively charged organic part and complex tetrachloroferrate, sometimes with partially exchanged chlorine atoms with bromine ones. It should be emphasized, that the synthesized MILSs are excellently soluble in water and methanol at room temperature, up to concentrations exceeding 10 mg/L, irrespectively to alkyl chain length. On the other hand, cetyltrimethylammonium bromide is characterized by limited aqueous solubility (Krafft point equal to 25 °C) while cetyltrimethylammonium–dysprosium­(III) monobromotrichloride forms precipitate from its methanolic solution even at room temperature.^20^ Crystallization of Mag-D-surfs yielded the formation of more complex, asymmetric compounds comprising, statistically, one FeCl_*x*_Br_4–*x*_^–^ and one Cl^–^ or Br^–^ counterion. Cl to Br ratio varied for particular compounds from 4.13 (C_10_-D_C_NMe_3_Mag) to 7.04 (C_12_-D_C_NMe_3_Mag). Such effects may be connected with the combination of spatial packing for shorter and longer alkyl chains with overall hydrophobicity of particular compounds. It should be emphasized, that the additional crystallization step enables the formation of more regular structures due to better spatial arrangement and possible enrichment of the solid phase with Cl. Moreover, similarly to the synthesized MILSs, our Mag-D-Surfs are freely soluble in water and methanol up to concentrations exceeding 20 mg/mL.

### Structure Elucidation and Thermal Behavior

The ESI-MS and FT-IR techniques, as well as Raman spectroscopy, are frequently used to characterize the chemical structures of particular products, especially when combining organic (surface active cation) and inorganic (complex counterion) counterparts.^14–16^ For covalent bonding within inorganic structures, such techniques may be supported by far-IR spectroscopy in the 40–500 cm^–1^ region. Those measurements are rather qualitative, as they indicate the presence of appropriate structural elements or the appearance of particular ionized fragments at the given *m*/*z* (mass-to-charge) ratio. The presence of impurities, especially larger amounts, may be signaled by appearing signals not fitting those assigned to the main structure, although it is difficult to even estimate their concentration. Thus, it is necessary to support such analyses with appropriate quantitative methods, such as XRF with an internal standard, direct elemental analysis, or ICP (inductively coupled plasma) for metal concentration. The combination of qualitative and quantitative methods enables the confirmation of crucial data, especially the molecular mass, a critical parameter for any further investigations. Such a complex approach is particularly important for magnetic surfactants, since even slight changes, e.g., a change in the coordination number of the counterion, can significantly affect their properties.

Spectroscopic analyses confirmed the chemical structures of the synthesized Mag-D-Surfs. Strong signals at 2800–3100 cm^–1^ and 1350–1500 cm^–1^ in FT-IR and Raman spectra revealed the presence of aliphatic hydrocarbon motifs, contributing to −CH_2_ and −CH_3_ groups—see Table 2, as well as Table S1 for single-tail single-head compounds and Figures S5 and S6 in the Supporting Information. Signals at around 330–340 cm^–1^ in Raman spectra revealed the presence of Fe–Cl bonds in magnetic counterions. ESI-MS spectra confirmed the presence of positively charged ionized amphiphilic motifs (C_*n*_H_2*n*+1_NBr^+^), attributed to appropriate alkyl chain lengths. The chemical composition of the studied compounds was determined by C, H, N, and Cl assay elemental analyses (see Tables 2 and S1). Survey XRF scans revealed the presence of only Cl, Br, and Fe heavy atoms, indicating the absence of incidental impurities such as sulfur, magnesium, or potassium associated with traces of reagents, drying agents, or other materials. The use of appropriate internal standards enabled quantitative estimation of the abundances of chlorine, bromine, and iron in the studied Mag-D-Surfs, as well as in linear magnetic ionic liquid surfactants, thereby confirming their structures and purities (see Table S2 in the Supporting Information). It should be noted that, despite the low accuracy of quantitative XRF, the method confirmed the preponderance of Cl over Br in all samples as well as Fe/Br ratios below 1.

**Table 2:** Spectroscopic Data and Selected Thermal Properties of the Studied Magnetic Dicephalic Surfactants^a^

					Elemental analyses							
magnetic surfactant	FT-IR (σ, cm^–1^)	Raman (σ, cm^–1^)	FIR (σ, cm^–1^)	ESI-MS [M]^+^ (calcd)	%C	%H	%N	%Cl	*T*_m_ (°C)^a^	–Δ*H*_m_ (J/g)^a^	*T*_cr_ (°C)^b^	Δ*H*_cr_ (J/g)^b^
C_10_-D_C_NMe_3_Mag	2922.95 (υ_as_C–H), 2853.07 (υ_s_C–H), 1478.40 (CH_2 bending_), 1376.66 (CH_3 bending_)	3105.49 (N^+^-CH_3_), 2911.88 (C–CH_3_), 1458.14 (CH_3_), 1301.76 (CH_2_), 965.10 (N^+^-CH_3_), 879.57 (CH_2_), 774.09 (N^+^-CH_3_), 546.49 (C–C), 369.18 (Fe–Cl)	391.00 (Fe–Cl and Fe–Br), 350.02 (FeCl_3_Br^–^ and FeCl_2_Br_2_^–^), 289.76 and 266.13 (Fe–Br), 247.81 and 221.30 (FeCl_*x*_Br_4–*x*_^–^), 227.56 (Br–Fe–Br), 139.33 (Cl–Fe–Cl), 126.80 (Br–Fe–Br), 121.50 (FeCl_*x*_Br_4–*x*_^–^), 110.89 and 74.73 (Br–Fe–Br)	379.3 (379.3)	39.5	7.70	4.86	24.7	146.1	26.57	118.2	21.37
C_12_-D_C_NMe_3_Mag	2923.65 (υ_as_C–H), 2852.29 (υ_s_C–H), 1482.61 (CH_2 bending_), 1376.66 (CH_3 bending_)	3035.49 (N^+^-CH_3_), 2884.04 (C–CH_3_), 1444.33 (CH_3_), 1301.83 (CH_2_), 965.90 (N^+^-CH_3_), 878.95 (CH_2_), 772.23 (N^+^-CH_3_), 554.43 (C–C), 332.36 (Fe–Cl)	390.04 (Fe–Cl and Fe–Br), 368.34 and 350.51 (FeCl_3_Br^–^ and FeCl_2_Br_2_^–^), 289.76 and 266.13 (Fe–Br), 246.85 and 222.26 (FeCl_*x*_Br_4–*x*_^–^), 227.80 (Br–Fe–Br), 139.33 (Cl–Fe–Cl), 121.01 (Br–Fe–Br), 99.80 and 73.28 (Br–Fe–Br)	407.3 (407.3)	42.8	8.22	4.75	26.3	148.7	37.28	124.4	15.28
C_14_-D_C_NMe_3_Mag	2955.30 (υ_as_C–H), 2886.00 (υ_s_C–H), 1485.90 (CH_2 bending_), 1393.27 (CH_3 bending_)	3016.67 (N^+^-CH_3_), 2955.01 (C–CH_3_), 1456.70 (CH_3_), 1291.55 (CH_2_), 968.68(N^+^-CH_3_), 902.68 (CH_2_), 758.72 (N^+^-CH_3_), 552.93 (C–C), 414.60 (Fe–Cl)	394.38 and 388.11 (Fe–Cl and Fe–Br), 350.02 (FeCl_3_Br^–^ and FeCl_2_Br_2_^–^), 289.76 and 266.13 (Fe–Br), 247.81 and 221.78 (FeCl_*x*_Br_4–*x*_^–^), 139.33 (Cl–Fe–Cl), 126.80 (Br–Fe–Br), 121.50 and 110.89 (Br–Fe–Br), 100.28 and 73.28 (Br–Fe–Br)	435.3 (435.3)	44.6	8.48	4.53	24.9	156.4	46.41	137.7	16.64

a See Figure S5 (FT-IR spectra), Figure S6 (Raman spectra), Figure S7 (FIR spectra), and Figures 1 and S8 and S9 (TGA and DSC curves).

b Heating rate, 10 K/min; endset.

c Heating rate, −10 K/min; onset.

In order to elucidate structural characterization of magnetic function bearing inorganic motifs, i.e., FeCl_*x*_Br_4–*x*_ complexes, far-IR spectra were recorded, utilizing Nujol mulls for wavenumbers 40–500 cm^–1^. According to ref 33, there exist several differences between stretching and bending modes of Fe–Cl and Fe–Br bonds, in particular FeCl_4_^–^, FeCl_3_Br^–^, FeCl_2_Br_2_^–^, FeClBr_3_^–^, and FeBr_4_^–^ complex ions. In general, signals at around 385–395 cm^–1^ are attributed to γ­(Fe–Cl) and γ­(Fe–Br) in all the studied specimens, while the band at around 345 cm^–1^ is present only in FeCl_3_Br^–^ and FeCl_2_Br_2_^–^ ions. Bands around 295 and 270 cm^–1^ are characteristic of γ­(Fe–Br) vibrations in mixed chlorobromoferrates. δ­(Cl–Fe–Cl) provides signals at around 135 and 115 cm^–1^, while δ­(Br–Fe–Br) in FeCl_2_Br_2_^–^ and FeClBr_3_^–^—at 120 and 74 cm^–1^. δ­(Br–Fe–Br) vibrations at 98 cm^–1^ are characteristic for FeBr_4_^–^ complex. Moreover, additional signals at around 268, 244, and 222 cm^–1^ are present in mixed chlorobromoferrates. On the other hand, density functional theory (DFT) simulations showed frequencies for FeCl_4_^–^ and FeCl_3_Br^–^ at 119 and 362 cm^–1^ as well as at 110, 235, 325, and 362 cm^–1^, respectively.^34^ For the studied Mag-D-Surfs (see Table 2 and Figure S7), as well as the standard linear MILSs (see Table S1 and Figure S7), nearly all the aforementioned vibrations are present in their far-IR spectra, with a possible exception for 98 cm^–1^ (signal at ca. 100 cm^–1^ may not be clearly distinguished as δ­(Br–Fe–Br) vibrations in FeBr_4_^–^). Our findings indicate that in the solid state, all or nearly all (presence of FeBr_4_^–^ is disputable) possible FeCl_*x*_Br_4–*x*_ complexes have been confirmed. Such findings are consistent with the facile exchange of Cl and Br atoms in solution and in crystalline structures. Possibly low concentration/lack of FeBr_4_^–^ complexes may arise from a Cl/Br ratio equal to 3:1 in raw materials.

Thermogravimetry (TGA) and differential scanning calorimetry (DSC) constitute complementary techniques to study materials’ and compounds’ thermal behavior, particularly with respect to chemical or physical degradation and phase behavior.^14–16^ In general, for optimal performance, magnetic surfactants should withstand elevated temperatures, especially in terms of irreversible chemical degradation.^15^ On the other hand, phase transition temperatures provide valuable information about their structure.^14,16^ According to TGA measurements (see Figures 1 and 2 as well as Figures S8–S11 in the Supporting Information), all studied Mag-D-Surfs surfactants exhibited thermal stability up to approximately 180 °C, so above melting points, while MILSs—even up to 220 – 250 °C. For C_*n*_-D_C_NMe_3_Mag very slight weight loss (not exceeding ca. 1%) is observed at ca. 160 °C, while the mass loss up to 200 °C was around 10%, indicating the start of thermal decomposition. Around 70% of the initial mass was lost up to 420 °C, most possibly due to the degradation of organic matter during heating (e.g., via Hoffmann degradation). Our MILSs surfactants (see Figures 2, S10, and S11) can withstand temperatures exceeding 180 °C without signs of any decomposition, while their thermal stability significantly depends on the alkyl chain length: C_12_–NMe_3_Mag is stable up to 250 °C, whereas C_16_–NMe_3_Mag is stable only up to 180 °C. For all C_*n*_-NMe_3_Mag 70% weight loss is gained at around 360 °C. In general, the lower temperature of initial decomposition for Mag-D-Surfs when compared with linear MILSs may be attributed to methine (CH) motifs in dicephalic C_n_-D_C_NMe_3_Mag surfactants, particularly vulnerable to breaking upon heating. On the other hand, the temperature needed to achieve 70% of the initial mass loss is higher for Mag-D-Surfs (ca. 420 °C) than for MILSs (around 360 °C) most possibly due to the higher percentage of inorganic matter for C_*n*_-D_C_NMe_3_Mag. It should be emphasized that the introduction of halogenoferrate counterion significantly improves cationic surfactant thermal stability: for example, cetyltrimethylammonium bromide fragmentation of the hexadecyl alkyl chain, yielding shorter derivatives, occurs at relatively low temperatures (250–300 °C).^35^ First of all, our DSC measurements comprise varying cooling rates in order to show any changes in subsequent heating curves, indicating (taking into consideration samples’ thermal stability confirmed by TGA) reversible processed occurring in samples. Due to obvious reasons (samples inertia) cooling curves, especially with the fastest rates, cannot be studied in this way—therefore only one first heating/cooling cycle (10 and 4 K/min) may provide any comparable enthalpies and phase transition temperatures. The DSC measurements (see Tables 1 and 2) confirmed melting points by the Boetius table with an accuracy of 1–2 °C, regardless of heating ratio (4 or 10 K/min), with the exception of C_14_-D_C_NMe_3_Mag (relative difference of ca. 5 °C), most possibly due to larger inertia of the system with the longest alkyl chain. In general, linear (single-headgroup single-tail)^16^ and gemini^14^ magnetic surfactants, comprising exclusively hydrocarbon-based structure (with exceptions for quaternary nitrogen and counterion), are characterized by very low melting points (in the range of 30–70 °C), increasing with lengthening of the alkyl chain. It has been postulated that such behavior is associated with weak interactions between [FeCl_*x*_Br_4–*x*_]^−^ and the quaternary ammonium headgroup.^14^ The appearance of a double melting peak in a DSC diagram is usually associated with the material heterogeneity, including amorphous/polycrystalline, two types of crystals, etc. In the present case, two possible forms, amorphous and polycrystalline, can appear at a given temperature and switching between them is possible. This switching behavior is governed by differences in cooling rates (see Figure 3). Taking this phenomenon into account we planned specific measurement programs, comprising different cooling rates, in contrast to standard measurement procedure, providing elimination of sample’s thermal history during the first cycle. However, in our case, this history is crucial for the final material’s state. In contrast to linear MILSs and magnetic gemini surfactants, our Mag-D-Surfs exhibit melting points exceeding 140 °C (the maximal value above 150 °C was determined for the compound with the longest alkyl chain—C_14_-D_C_NMe_3_Mag). Such behavior may indicate stronger interactions between quaternary ammonium headgroups and counterions produced by their close-packing due to their geometric proximity (two positively charged motifs separated by three carbon atoms placed at one side of a long alkyl chain).^14^ Moreover, it is beneficial for the further applicability of Mag-D-Surfs, as they can occur as solids over a wide range of ambient temperatures and resolidify after melting without risk of decomposition. The broadness of Mag-D-Surfs melting peaks may be explained by its structural complexity, so it appears to be distributed over a temperature range, rather than a single sharp thermodynamic event. A Mag-D-Surfs sample is not uniform at the molecular level (different crystallite sizes, different degrees of perfection, etc.) so each crystalline region melts at a slightly different temperature. The enthalpies of melting (−Δ*H*_m_) for our studied Mag-D-Surfs are increasing with the lengthening of the alkyl chain, although such behavior is not observed for the enthalpies of crystallization (Δ*H*_cr_). Values of −Δ*H*_m_ do not exceed ca. 50 J/g, what indicates the reversible character of this transition—for example, the enthalpy of octadecyltrimethylammonium bromide transition into the disordered phase is over 140 J/g (105.47 °C), while CnTAB surfactants are known for thermal instability.^35,36^ The composition, i.e., the dominance of crystalline or amorphous phase in a particular Mag-D-Surf, may be controlled by appropriate thermal treatment of the sample. To assess key parameters of these processes, we performed appropriate DSC/TGA (see Figures 1, S8, and S9) and polarization microscopy experiments (see Figure 3). Moreover, similar sets of experiments were used to study our MILSs surfactants in order to show their thermal behavior (see Figures 2, 4–6, S10, and S11).

**Figure 1:**
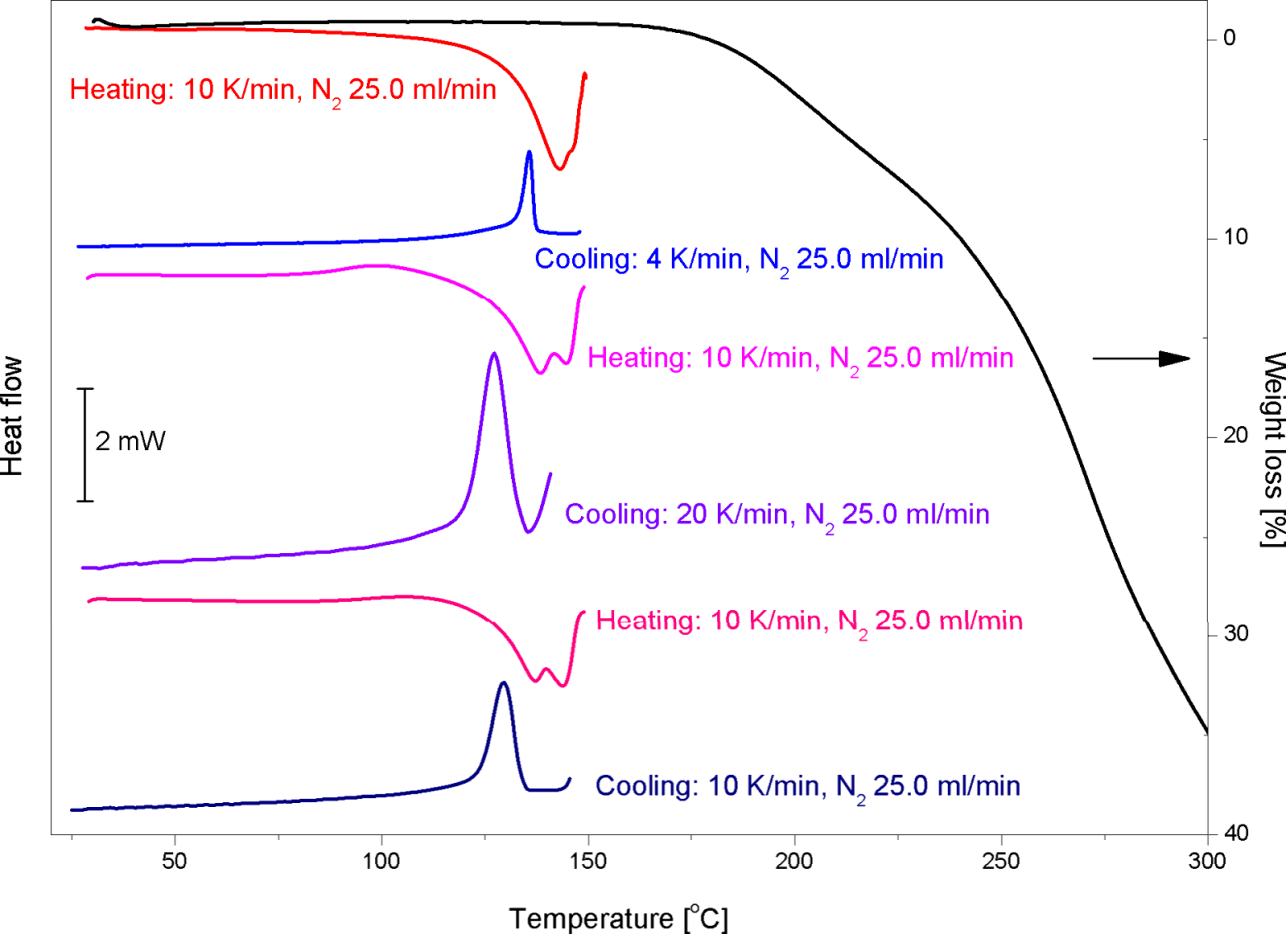
TGA (*m* = 6.0000 mg) and DSC (*m* = 5.4500 mg) curves for C_12_D_C_NMe_3_Mag. Cf. Table 2.

**Figure 2:**
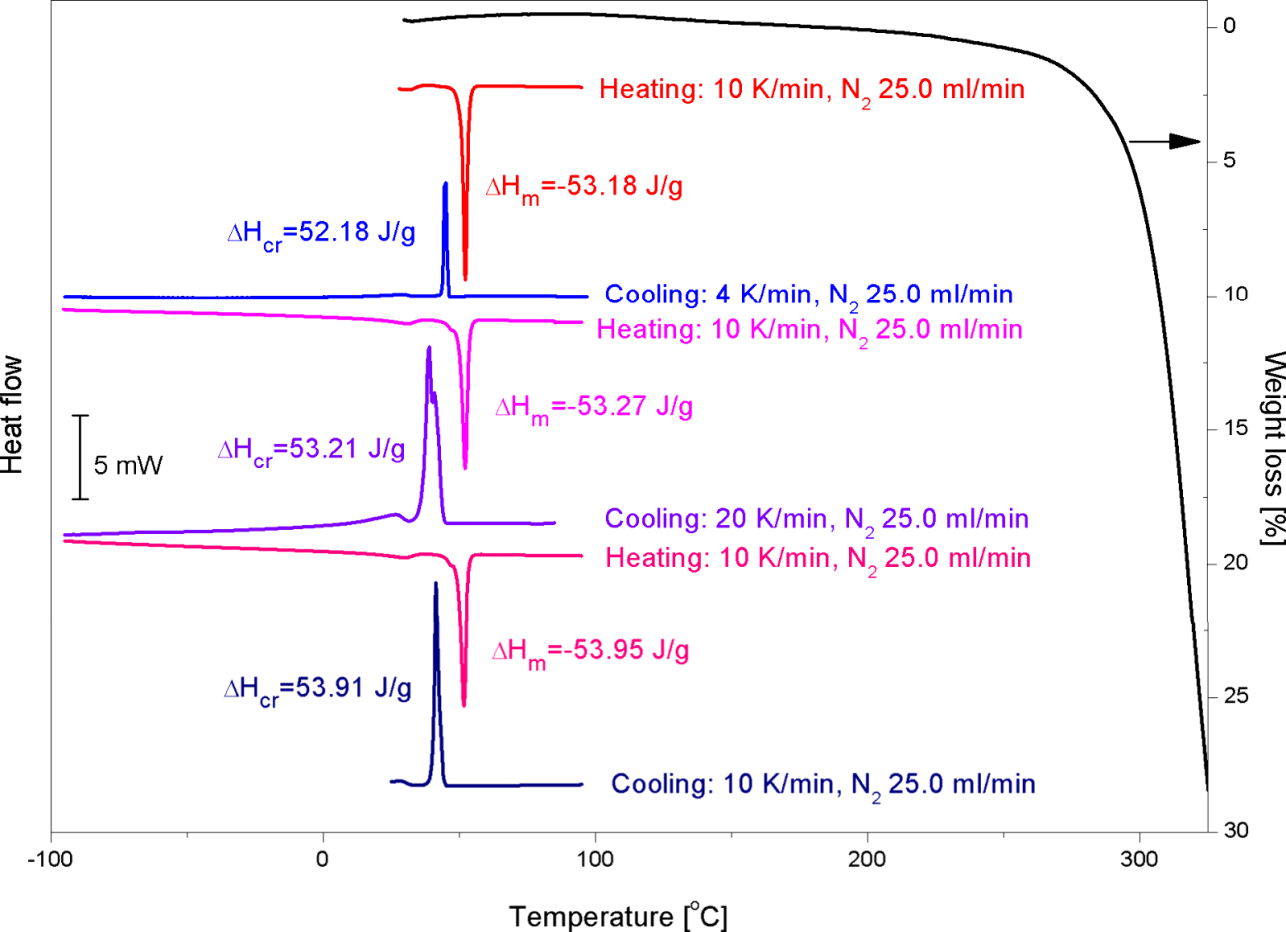
TGA (*m* = 8.5460 mg) and DSC (*m* = 2.7080 mg) curves for C_14_NMe_3_Mag. Cf. Table S1.

**Figure 3:**
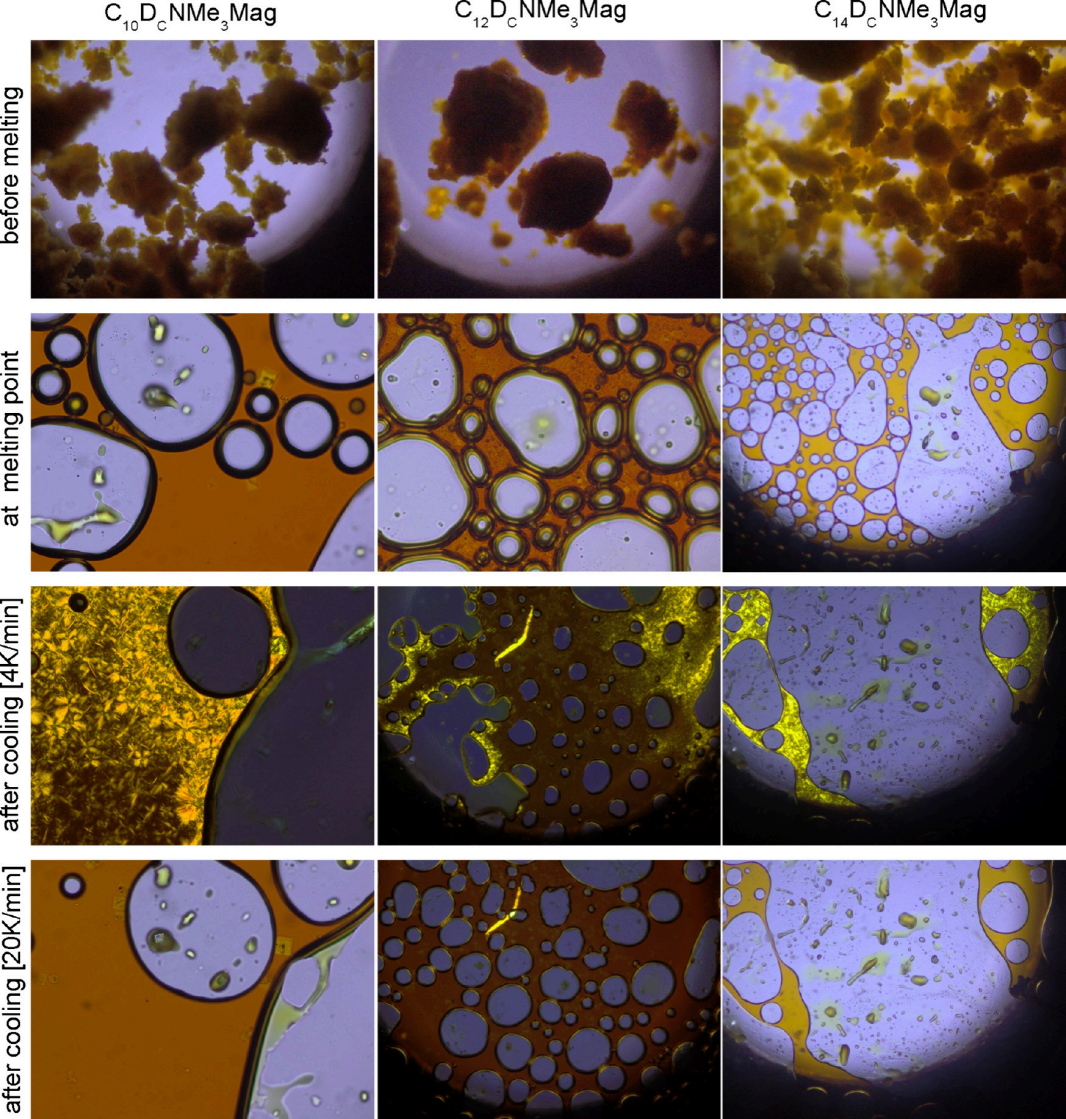
Polarization micrographs for surfactants C_*n*_-D_C_NMe_3_Mag (*n* = 10, 12, 14) under the studied conditions.

**Figure 4:**
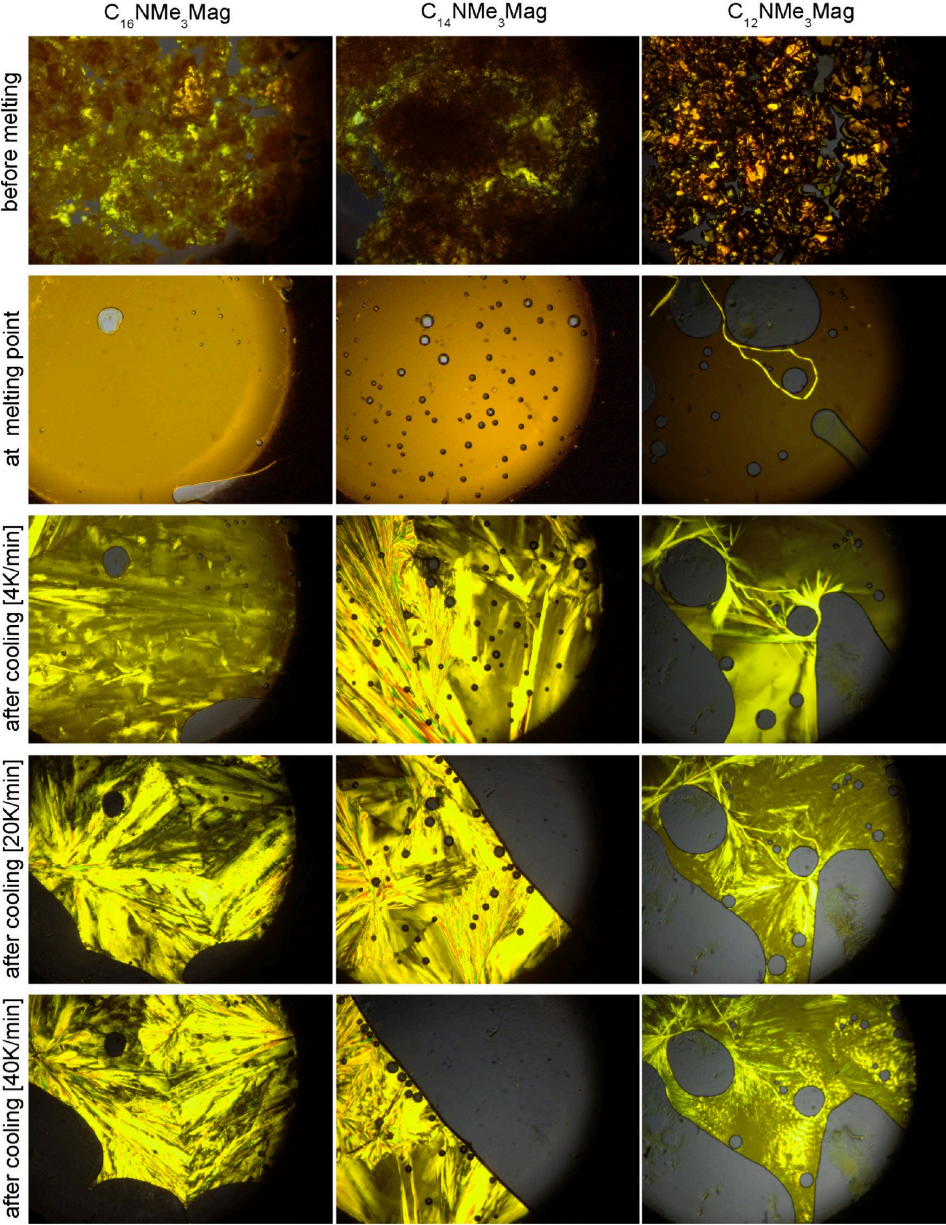
Polarization micrographs for MILSs C_*n*_-NMe_3_Mag (*n* = 12, 14, 16) under the studied conditions.

**Figure 5:**
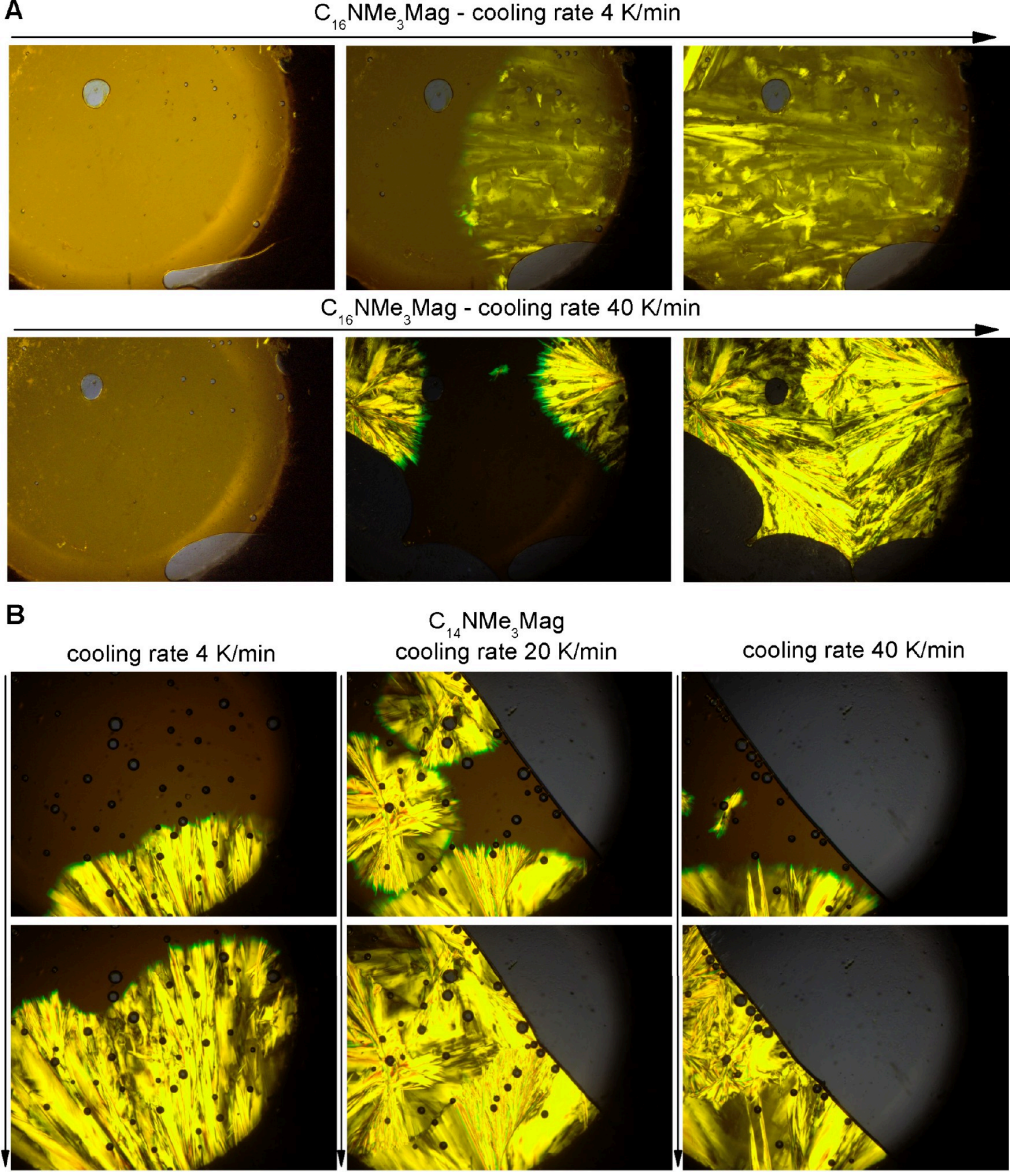
Formation of anisotropic phases for C_16_–NMe_3_Mag (A) and C_14_–NMe_3_Mag (B) cooling with varying rates.

**Figure 6:**
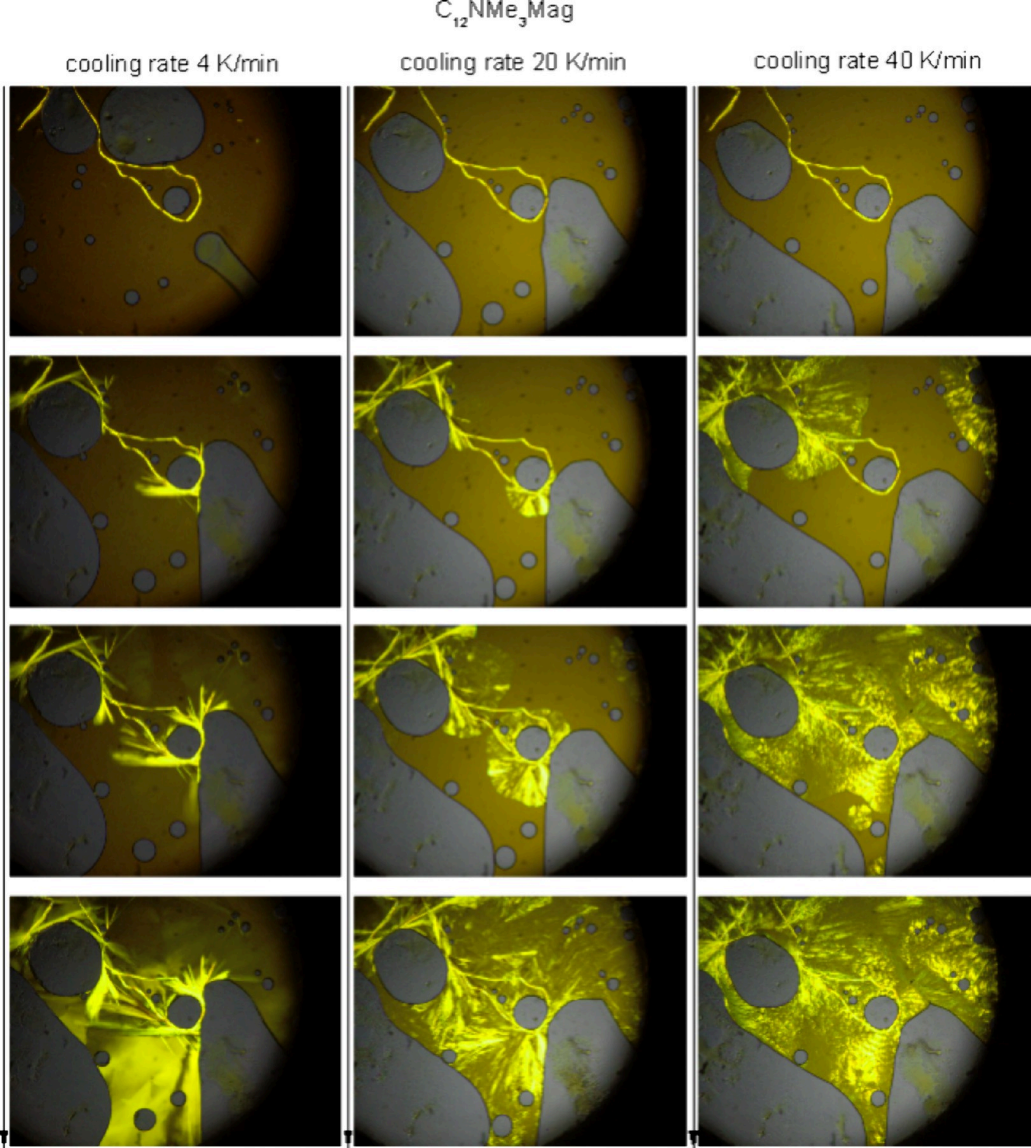
Formation of anisotropic phases for C_12_–NMe_3_Mag cooling with varying rates.

Mag-D-Surf samples were heated up to 155 °C (in order to achieve melting without any loss of weight) and cooled with varying rates: 4, 10, or 20 K/min, following appropriate programs. The polarization microscopy images taken at slow cooling from around 155 °C to room temperature showed the formation of crystalline phases, especially intensive below around 135 °C, consistent with the exothermal peak in the DSC curve. On the other hand, fast cooling allowed for “freezing” amorphous structure, and no visible crystallites (polarization microscopy, magnification 30×) were observed during cooling to room temperature or even below. The characteristic feature of DSC curves is the absence of a glass transition below the melting point, although for C_10_-D_C_NMe_3_Mag an endothermic peak at ca. 85 °C was observed during the first heating cycle. Such behavior was unique only for this particular compound with the shortest alkyl chain and may be attributed to solid phase transition, known for some ionic surfactants.^27^ On the other hand, MILSs surfactants behave as standard crystalline compounds with melting and crystallization peaks with enthalpies irrespective of heating or cooling rates, respectively (see Figures 2, S10, and S11). For MILSs (see Table S1) their enthalpies of melting (−Δ*H*_m_) and crystallization (Δ*H*_cr_) are increasing with the lengthening of the alkyl chain, while their absolute values are nearly the same for particular compounds. Such behavior clearly indicates complete reversibility of both processes without any signs of chemical decomposition or changes in phase structure, e.g., amorphous/crystalline parts ratio.

In order to confirm a lack of any changes in chemical structure for the studied compounds of both linear (single-chain single-headgroup) and dicephalic architecture, additional FT-IR studies have been performed. For C_14_–NMe_3_Mag (MILS) and C_12_-D_C_NMe_3_Mag (Mag-D-Surf) derivatives, the FT-IR spectra (see Figure S12) were recorded before and after melting and solidification. The obtained data clearly show no changes in any spectral regions, including characteristic bands of hydrocarbon motifs at ca. 2750–3000 cm^–1^ as well as the fingerprint region, thus confirming thermal stability of the studied compounds to at least their melting points at ca. 55 and ca. 150 °C for MILS and Mag-D-Surf, respectively.

The unique behavior of C_*n*_-D_C_NMe_3_Mag surfactants may be explained by a subtle balance between ionic interactions, arising from charged headgroups and counterions, and van der Waals forces, which drive the stacking of hydrophobic parts together. The crucial factor, responsible for solidification upon cooling into isotropic and anisotropic structures, is the combination of the amphiphilic structure and the tendency to form spherical initial crystals.^27^ The growth of the crystalline phase from such micelle-like nucleus structures may be interrupted by antiphase (i.e., boundary phase separating initial crystals) plasticity and viscosity. Therefore, it is possible to control crystalline-phase growth; the formation of anisotropic structures may be terminated at the initial step, yielding single micelle-like structures.^27^ It should be emphasized that the balance between forces, playing a significant role in such behavior, is very subtle and strongly dependent on the dimensions of particular counterparts (charged amphiphilic cation and negatively charged counterions). Recalling, only for C_10_-D_C_NMe_3_Mag, a significant number of regular subphases of increased thermal resistance is observed, while MILSs (single-tail single-headgroup surfactants) are characterized by melting points below 75 °C. Moreover, appropriate strength–dimension balance may be achieved only for halogenoferrate counterions, enabling their melting within the thermal stability region (i.e., below around 175 °C).^14,26^ On the other hand, MILSs form anisotropic structures upon cooling their melts at any rate (4, 20, or even 40 K/min)—see Figure 4. Formation of crystalline phases constitutes a very rapid process with a clearly visible “crystallization front”—see Figures 5 and 6. In general, the formation of crystalline structures initiates within the boundaries of the liquid phase, although for C_12_–NMe_3_Mag some spherulites within the bulk melt are also observed (see Figure 6).^37^ The thermal behavior of Mag-D-Surfs opens the possibility to design devices utilizing phase change memory (PCM) effects. Systems of this type require materials whose phases, with different physicochemical properties, can coexist at a single temperature.^38^ One phase is stable, and the other is metastable. The key is the ability to convert one phase to another in a controlled, reversible manner. The switching factor is the rate of temperature change, controlled by the width of the electric current pulse applied to the memory element. All the aforementioned abilities are characteristic of our new Mag-D-Surfs at convenient temperature ranges. It should be emphasized that our studies clearly show that controlled coexistence of isotropic and anisotropic phases is distinctive only for Mag-D-Surfs but not for MILSs.

### Magnetic Susceptibility

In order to elucidate the magnetic properties of surfactants in bulk (solid) state, typically SQUID magnetometry measurements of powdered samples were performed for a variable magnetic field at a temperature of 300 K.^14,16^ Magnetization measurements enable gaining information about paramagnetic behavior as well as the effective magnetic moment. On the other hand, SQUID magnetometry provides direct detection of Fe­(III) in magnetic surfactants. Recalling, our Mag-D-Surfs comprise a new group of magnetic surfactants in relation to those recently studied in literature, especially magnetic ionic liquid surfactants^16^ and gemini magnetic surfactants,^14^ characterized by significantly different thermal and phase behavior despite some structural similarities (see section Structure Elucidation and Thermal Behavior).

The theoretical values of the effective magnetic moments of the Fe­(III) ion, with *n* = 5 unpaired electrons, were calculated according to the equation:

$$\mu_{\mathrm{so}}=\sqrt{n\left(n+2\right)}=5.92B.M.$$

The values of the experimental and theoretical effective moments for both the studied novel Mag-D-Surfs and the standard magnetic ionic liquid surfactants synthesized by us for comparison are presented in Table 3. Foremost, there is an excellent agreement of theoretical (μ_so_) and experimental (μ_eff_) values for magnetic ionic liquid surfactants, indicating their purity and confirming the effectiveness of our synthetic approaches. On the other hand, for our newly devised Mag-D-Surfs, similar values of μ_eff_ (around 5–6) were obtained. Such findings are consistent with their chemical composition by elemental analyses, clearly indicating the presence of one Fe­(III) complex per alkyl chain (see data in Table 3). In general, only one compound, C_10_-D_C_NMe_3_Mag, exhibited slightly lower (when compared with its analogues with longer alkyl chains) values of μ_eff_ and magnetic susceptibility (χ_g_). Its behavior may be associated with specific phase structure (presence of regular subphases of increased thermal resistance and additional endothermic peak at ca. 85 °C for heating—see section Structure Elucidation and Thermal Behavior for details), comprising possibly nonmagnetic substructures.

**Table 3:** Magnetic Properties of the Studied Magnetic Dicephalic Surfactants and Linear Magnetic Ionic Liquid Surfactants

			magnetic moment (μ_eff_) in B.M.	
magnetic surfactant and chemical composition^a^	*M* ^a^ (g/mol)	χ_g_ × 10^5^ (emu/g)	exptl (μ_eff_)	theor (μ_so_)
Mag-D-Surfs (Melting Point > 100 °C)				
C_10_-D_C_NMe_3_Mag	577.039	1.86	5.15	5.92
C_19_H_44_Br_0.974_Cl_4.026_FeN_2_				
C_12_-D_C_NMe_3_Mag	589.453	2.16	5.54	
C_21_H_48_Br_0.622_Cl_4.378_FeN_2_				
C_14_-D_C_NMe_3_Mag	619.113	2.08	5.64	
C_23_H_52_Br_0.658_Cl_4.342_FeN_2_				
MILSs (Melting Point < 100 °C)				
C_12_–NMe_3_Mag	447.614	3.06	5.78	5.92
[DTA][FeCl_*x*_Br_4–*x*_^–^] C_15_H_34_Br_0.483_Cl_3.517_FeN				
C_14_–NMe_3_Mag	463.851	2.81	5.65	
[TTA][FeCl_*x*_Br_4–*x*_^–^] C_17_H_38_Br_0.217_Cl_3.783_FeN				
C_16_–NMe_3_Mag	482.265	2.61	5.56	
[CTA][FeCl_*x*_Br_4–*x*_^–^] C_19_H_42_Cl_4_FeN				

a Calculated according to the results of elemental analyses.

The studied magnetic dicephalic surfactants, similar to standard (linear) magnetic ionic liquid surfactants (see Figure 7 and Table 3), exhibited purely paramagnetic behavior with no magnetization hysteresis at room temperature.^15,16^ In general, SQUID magnetometry confirmed the presence of paramagnetic Fe­(III) ions at a 1:1 molar ratio (surface-active cation/Fe^3+^) for both magnetic ionic liquid surfactants and our newly devised Mag-D-Surfs.

**Figure 7:**
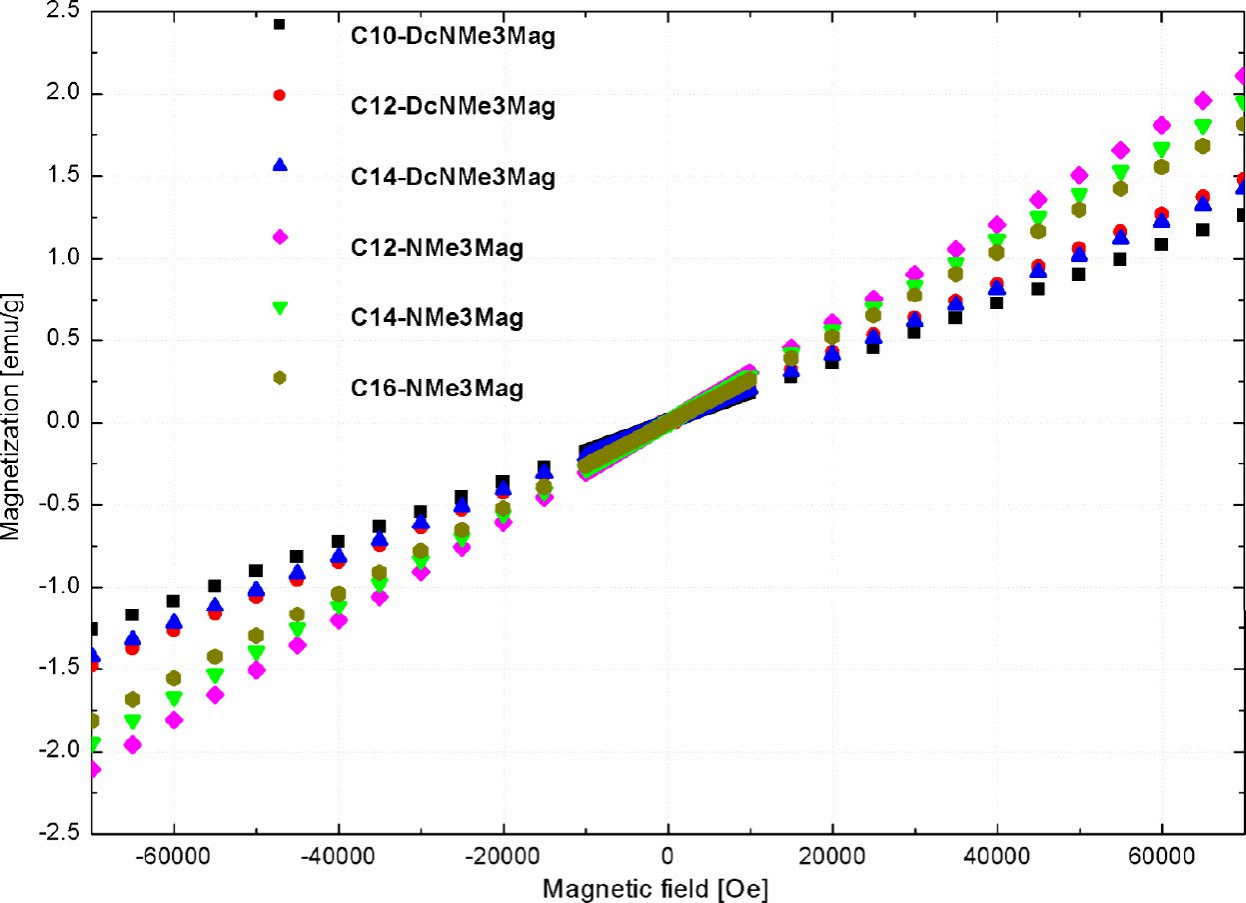
Magnetization of the studied magnetic dicephalic surfactants and standard magnetic ionic liquid surfactants (MILSs) as a function of applied magnetic field at 300 K.

### Surface Activity

The characteristic behavior of magnetic surfactants may constitute the dependence of surface tension on an external magnetic field, either uniform or gradient.^12,16,20^ Such a type of responsivity enables preparing unique types of smart materials, such as emulsions with magnetic field-triggered properties.^10^ Figure 8 illustrates the results of comparing surface tension isotherms of the Mag-D-Surfs with their dicephalic counterparts without the magnetic functionality, i.e., with two Br^–^ anions as counterions.^28^ Recalling, our investigations on surface tension measurements—focused on CMC determination—were conducted utilizing pendant drop method due to its simplicity and possibility for magnetic field-triggered measurements. On the other hand, we took into account the possibility of depletion of the surfactant, but this effect is known to affect only diluted solutions so, in our case all CMC values (exceeding 10^–3^ M) are determined accurately.^39^

**Figure 8:**
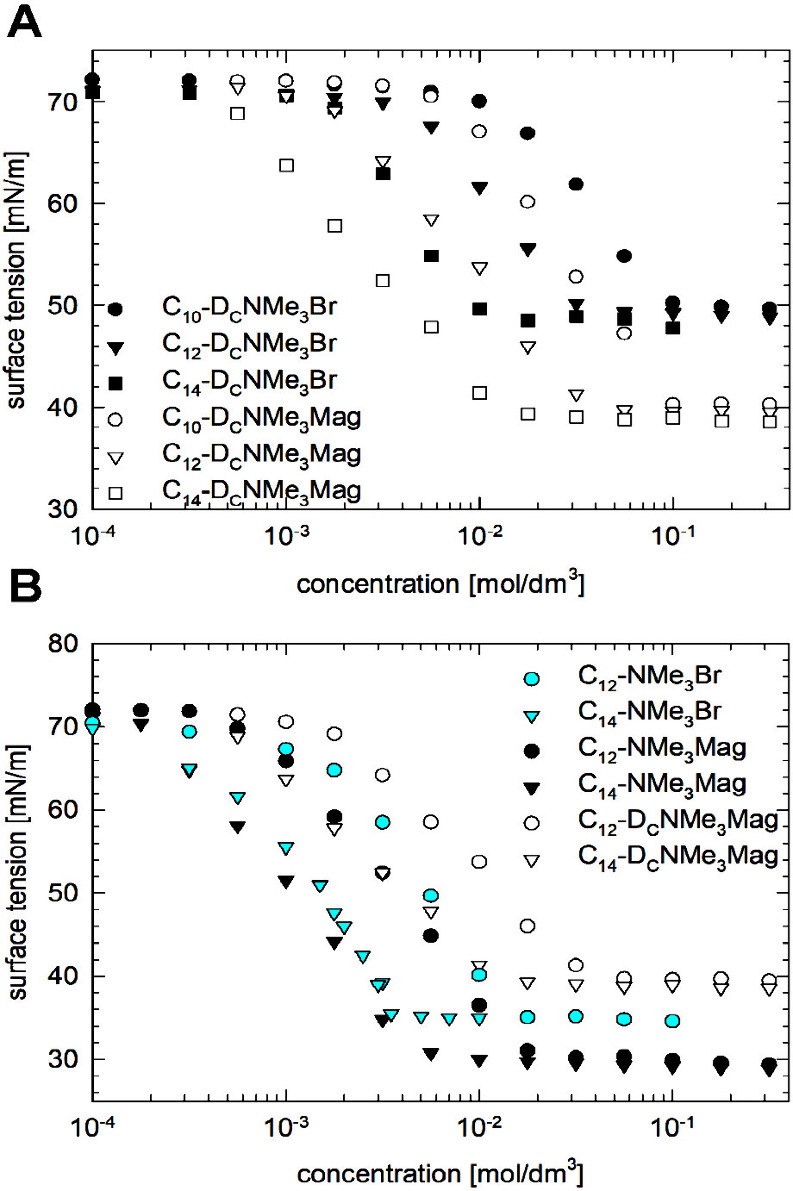
Dependence of surface tension on surfactant concentration for (A) the dicephalic magnetic surfactants and their counterparts without the magnetic functionality and (B) the chosen dicephalic magnetic surfactants (white symbols) and their linear counterparts with and without the magnetic functionality (black and blue symbols, respectively).

The addition of the magnetic functionality to the dicephalic surfactant by replacing the bromide counterion with ferrate increases the surface activity, shifts the CMC to lower surfactant concentrations, and decreases the surface tension above the CMC by approximately 10 mN/m. It results from more effective neutralization of surfactant charge at the interface by a more polarizable ferrate counterion.^40^ The same effect could be observed for the linear surfactants, as illustrated in Figure 8, where the dependencies of surface tension on surfactant concentration for the Mag-D-Surfs and their linear counterparts with the same alkyl chain lengths are additionally compared. The linear magnetic surfactants exhibit higher surface activity compared with the dicephalic ones as a result of their better solubility and weaker electrostatic repulsion at the interface between single-charged hydrophilic groups. In Table 4, the values of CMC for all studied magnetic surfactants obtained by surface tension measurements are collected together with the respective values for the surface tension at CMC. They illustrate the effect of the alkyl chain length on the surface activity of magnetic surfactants, which follows the Traube rule. For our studies, we have considered two types of surfactants with magnetic functionality: single-tail single-headgroup MILSs as well as single-tail double-headgroup Mag-D-Surfs. The CMC values for MILSs (C_n_-NMe_3_Mag) were slightly less but very similar to their nonmagnetic equivalents (C_*n*_-NMe_3_Br), i.e., DTAB (C_12_-), TTAB (C_14_-), and CTAB (C_16_-). The difference may originate from the higher polarizability of the ferrate counteranion. Mag-D-Surfs exhibit lower surface activity, i.e., higher CMC compared to that of MILSs, as a result of the stronger electrostatic repulsion at the interface between double-charged hydrophilic groups. This double charge is partially compensated by the formation of transient complexes with counterions.

**Table 4:** CMC Values of the Studied Magnetic Dicephalic Surfactants and Linear Magnetic Ionic Liquid Surfactants

surfactant	CMC (mmol/dm^3^)	surface tension at CMC (mN/m)
C_12_-NMe_3_Mag	17	31
C_14_-NMe_3_Mag	5	30
C_16_-NMe_3_Mag	1.2	29
C_10_-D_C_NMe_3_Mag	98	41
C_12_-D_C_NMe_3_Mag	35	40
C_14_-D_C_NMe_3_Mag	13	39

Figure 9A illustrates the effect of the uniform magnetic field of the maximal strength applied in the experiments (*B* = 650 mT) on the surface tension of Mag-D-Surf solutions measured by the drop shape analysis. The magnetic field was applied perpendicular to the droplet axis.

**Figure 9:**
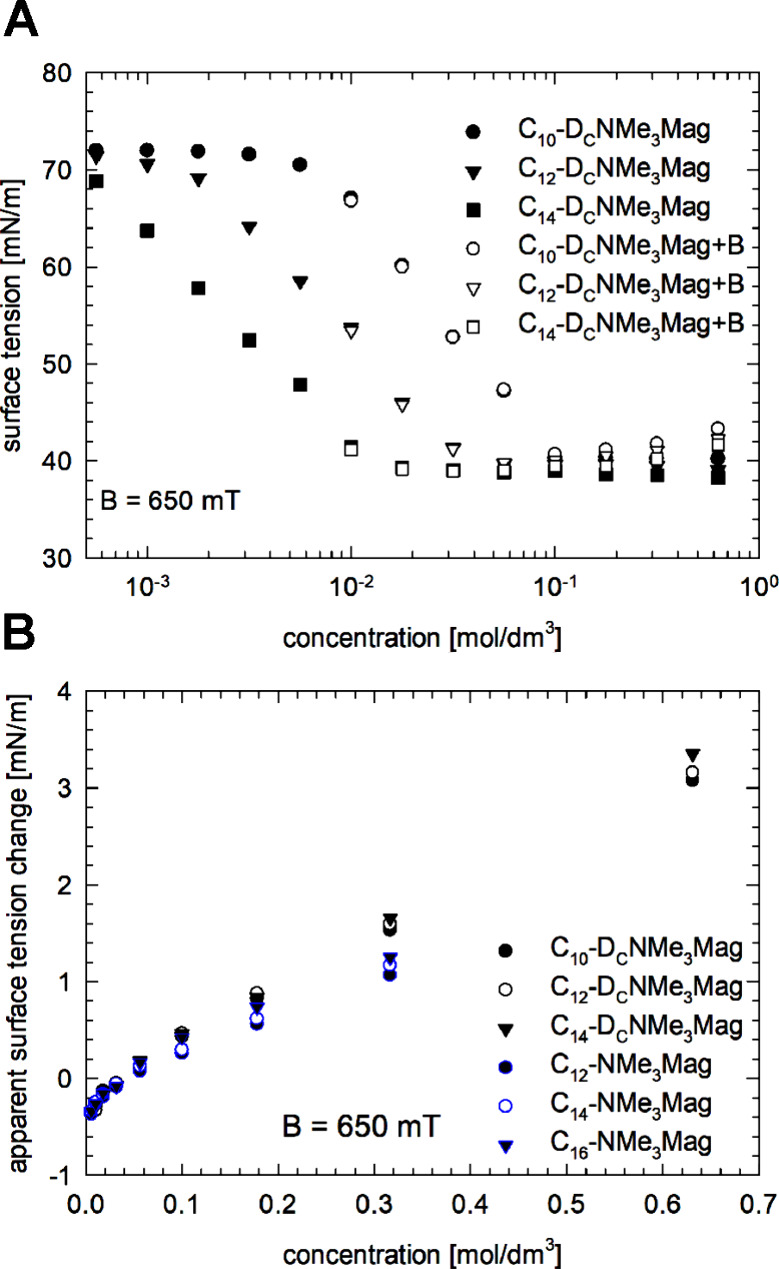
Effect of the applied uniform magnetic field (*B* = 650 mT) on the measured surface tension with respect to Mag-D-Surfs concentration (A); dependence of the apparent surface tension increase, due to the drop deformation in the presence of a magnetic field of 650 mT, on the concentration of magnetic surfactants (B).

Some positive increases in the measured surface tension were observed at concentrations well above the respective CMCs. Below the CMC, only a slight decrease in the observed surface tension, at the level of experimental error, could be noticed. A similar behavior was observed for linear magnetic surfactants, C_n_-NMe_3_Mag. Figure 9B illustrates the dependence of the apparent change in the surface tension of solutions of magnetic surfactants on a uniform magnetic field of 650 mT. In pure water and at low surfactant concentrations (approximately 50 mM), the measured surface tension is lower than that in the absence of the magnetic field. That is the result of the drop representing a diamagnetic medium. In a magnetic field, it tends to be expelled and becomes more elongated. Therefore, the apparent surface tension change was negative. Upon the addition of a paramagnetic component, the magnetic properties of the drop medium turn paramagnetic. Then, the drop is drawn into the magnetic field, its shape becomes more spherical, and the measured surface tension apparently increases. Since, as shown in Table 3, the magnetic moment values for all surfactants were similar, the threshold concentration for the diamagnetic-to-paramagnetic fluid transition is the same. The differences in the apparent surface tension increase at high surfactant concentrations as well as their deviation from a linear concentration dependence can be attributed to micellization, which occurs above the CMC for all surfactants.

## Conclusions

Custom-designed products characterized by magneto-responsivity and surface activity open up a wide range of potential applications in modern technologies. 2-Alkyl-*N*,*N*,*N*,*N*′,*N*′,*N*′-hexamethylpropan-1,3-ammonium halogenoferrates (C_*n*_-D_C_NMe_3_Mag; *n* = 10, 12, 14) represent a unique class of magnetic surfactants with a dicephalic (double-charge) structure possessing, in comparison to conventional structures, lower critical micelle concentration values. Their synthetic route made it possible to obtain ultrapure Mag-D-Surfs and MILSs under mild conditions for either preparation or purification. The studied C_n_-D_C_NMe_3_Mag surfactants in relation to linear magnetic ionic liquids surfactants—alkyltrimethylammonium halogenoferrates (alkyl: dodecyl [DTA]­[FeCl_*x*_Br_4–*x*_^–^], tetradecyl [TTA]­[FeCl_*x*_Br_4–*x*_^–^], and cetyl [CTA]­[FeCl_*x*_Br_4–*x*_^–^])—showed external magnetic field dependence of their aqueous solutions’ surface tension. Magnetometry confirmed purely paramagnetic behavior of all the studied surfactants with a 1:1 (surface active cation/Fe^3+^) molar ratio, while thermogravimetry, scanning calorimetry, and polarization microscopy showed the unique thermal behavior, opening the possibility of their performance as phase change memory (PCM) materials. It should be emphasized that only Mag-D-Surfs exhibit the possibility to control the phase structure by appropriate cooling rates, while MILSs not. A combination of the surface activity and ability to change the magnetic properties of liquid phases allows their application to form magnetically responsive foams and emulsions.

## Supplementary Material



## Notes

Additional experimental details; materials and methods, including spectroscopic data and selected thermal properties of the studied linear magnetic ionic liquids surfactants; estimated heavy atoms abundance by XRF; ^1^H NMR, FT-IR, Raman, and FIR spectra; DSC and TGA curves (PDF)
