# Supplementary material for: Asymmetrical Two-Headed Linear Cationic Surfactants with Halogenoferrate Magnetic and Bromide Counterions: Synthesis, Thermal Behavior, Magnetic Performance, and Surface Properties
Source: Langmuir. 2026 Jun 9;42(24):17298–312. doi: 10.1021/acs.langmuir.6c00730 (PMC13309002; doi:10.1021/acs.langmuir.6c00730)
Supplement: Supplementary file 1 [file la6c00730_si_001.pdf]

Supporting Information

Asymmetrical Two-Headed Linear Cationic Surfactants with Halogenoferrate Magnetic and Bromide Counter-Ions: Synthesis, Thermal Behavior, Magnetic Performance, and Surface Properties

Łukasz Lamch[a)], Maria Korabik[b)], Dawid Szarpak[a)], Przemysław Szklarz[b)], Mariusz Borkowski[c)], Piotr Warszyński[c)], Kazimiera A. Wilk[a)]*

[a)] Faculty of Chemistry, Wrocław University of Science and Technology, Wybrzeże Wyspiańskiego 27, 50-370 Wrocław, Poland

[b)] Faculty of Chemistry, University of Wrocław, F. Joliot-Curie 14, 50-383 Wrocław, Poland

[c)] Jerzy Haber Institute of Catalysis and Surface Chemistry, Polish Academy of Sciences, Niezapominajek 8, 30-239 Kraków, Poland

** Corresponding author*

E-mail address:

lukasz.lamch@pwr.edu.pl (Ł. Lamch)

Table of contents Page

1. Spectroscopic and thermal characterization of the studied linear magnetic ionic liquids surfactants. S3

2. Chemical composition by XRF. S4

3. ^{1}H NMR, FT-IR, Raman and FIR spectra of the studied compounds. S5

4. TGA and DSC curves for $C_{10}D_{C}NMe_{3}Mag$, $C_{14}D_{C}NMe_{3}Mag$, $C_{12}NMe_{3}Mag$ and $C_{16}NMe_{3}Mag$. S11

5. FT-IR spectra for C_{14}-$NMe_{3}Mag$ (MILS) and C_{12}-$D_{C}NMe_{3}Mag$ (Mag-D-Surf) before and after melting and solidification. S15

1. Spectroscopic and thermal characterization of the studied linear magnetic ionic liquids surfactants

Table S1. Spectroscopic data and selected thermal properties of the studied linear magnetic ionic liquids surfactants – see Figure S5 (FT-IR spectra), Figure S6 (Raman spectra), Figure S7 (FIR spectra) and Figures 2, S10 & S11 (TGA and DSC curves).

Magnetic surfactant	FT-IR (σ, cm^{-1})	Raman (σ, cm^{-1})	FIR (σ, cm^{-1})	ESI-MS $[M]^+$ (calc.)	Elemental analyses				T_m (°C)[a]	-ΔH_m (J/g)[a]	T_{cr} (°C)[b]	ΔH_{cr} (J/g)[b]
					%C	%H	%N	%Cl				
C_{12}-NMe_3Mag [DTA][$FeCl_xBr_{4-x}^-$]	2921.60 (υ_{as}C-H), 2852.67 (υ_sC-H), 1486.12 ($CH_{2\ bending}$), 1375.55 ($CH_{3\ bending}$)	3021.79 (N^+-CH_3), 2937.99 (C-CH_3), 1446.36 (CH_3), 1300.52 (CH_2), 965.10 (N^+-CH_3), 775.50 (N^+-CH_3), 590.33 (C-C), 333.62 (Fe-Cl)	375.09 (Fe-Cl and Fe-Br), 350.51 ($FeCl_3Br^-$ and $FeCl_2Br_2^-$), 289.76 and 266.13 (Fe-Br), 246.85 and 222.26 ($FeCl_xBr_{4-x}^-$), 227.08 (Br-Fe-Br), 139.82 (Cl-Fe-Cl), 126.80 (Br-Fe-Br), 121.50 ($FeCl_xBr_{4-x}^-$), 110.89 and 100.28 (Br-Fe-Br),73.28 (Br-Fe-Br)	228.3 (228.3)	40.2	7.67	3.13	27.6	36.93	35.69	-	25<
C_{14}-NMe_3Mag [TTA][$FeCl_xBr_{4-x}^-$]	2919.80 (υ_{as}C-H), 2851.49 (υ_sC-H), 1485.88 ($CH_{2\ bending}$), 1470.42 ($CH_{3\ bending}$)	3029.89 (N^+-CH_3), 2888.68 (C-CH_3), 1446.27 (CH_3), 1299.22 (CH_2), 974.69 (N^+-CH_3), 759.75 (N^+-CH_3), 610.12 (C-C), 351.44 (Fe-Cl)	393.41 11 (Fe-Cl and Fe-Br), 349.54 ($FeCl_3Br^-$ and $FeCl_2Br_2^-$), 289.28 and 265.65 (Fe-Br), 247.33 and 220.81 ($FeCl_xBr_{4-x}^-$), 227.08 (Br-Fe-Br), 138.85 (Cl-Fe-Cl), 126.32 (Br-Fe-Br), 121.01 and 110.41 (Br-Fe-Br), 99.80 and 74.25 (Br-Fe-Br)	256.3 (256.3)	44.0	8.27	3.02	28.9	53.61	53.18	43.45	53.91
C_{16}-NMe_3Mag [CTA][$FeCl_xBr_{4-x}^-$]	2955.30 (υ_{as}C-H), 2886.00 (υ_sC-H), 1485.90 ($CH_{2\ bending}$), 1393.27 ($CH_{3\ bending}$)	3028.00 (N^+-CH_3), 2888.00 (C-CH_3), 1452.00 (CH_3), 1320.00 (CH_2), 974.00 (N^+-CH_3), 910.00 (CH_2), 768.00 (N^+-CH_3), 582.00 (C-C), 336.00 (Fe-Cl)	393.41 (Fe-Cl and Fe-Br), 350.02 ($FeCl_3Br^-$ and $FeCl_2Br_2^-$), 289.76 and 266.13 (Fe-Br), 247.81 and 227.08 ($FeCl_xBr_{4-x}^-$), 221.78 ($FeCl_xBr_{4-x}^-$), 139.33 (Cl-Fe-Cl), 126.80 (Br-Fe-Br), 121.98 and 111.38 (Br-Fe-Br), 100.28 and 73.77 (Br-Fe-Br)	284.3 (284.3)	47.3	8.80	2.91	29.4	66.39	61.18	54.31	60.84

[a] Heating rate: 10 K/min, endset; [b] Heating rate: -10K/min, onset

2. Chemical composition by XRF

Table S2. Estimated heavy atoms abundance for the studied surfactants by XRF.

Magnetic surfactant	Cl:Fe	Br:Fe	Cl:Br
C_{12}-NMe_3Mag [DTA][$FeCl_xBr_{4-x}^-$]	4.563	0.583	7.832
C_{14}-NMe_3Mag [TTA][$FeCl_xBr_{4-x}^-$]	3.761	0.814	4.623
C_{16}-NMe_3Mag [CTA][$FeCl_xBr_{4-x}^-$]	3.927	0.834	4.707
C_{10}-$D_C NMe_3$Mag	4.335	0.504	8.597
C_{12}-$D_C NMe_3$Mag	3.702	0.564	6.559
C_{14}-$D_C NMe_3$Mag	4.281	0.484	8.835

3. ^{1}H NMR, FT-IR, Raman and FIR spectra of the studied compounds

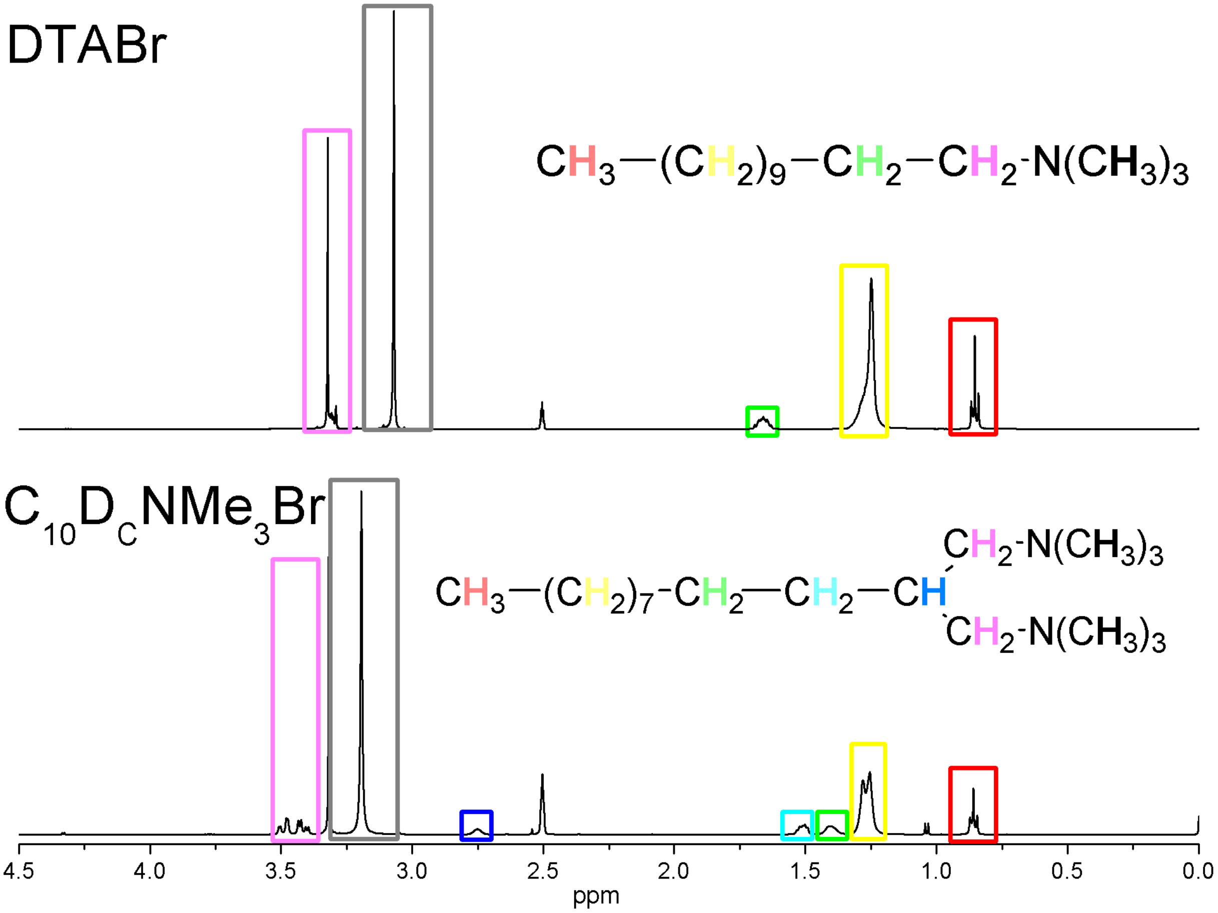

Figure S1. ^{1}H NMR spectra of parent non-magnetic surfactants: DTABr and $C_{10}D_{C}NMe_{3}Br$.

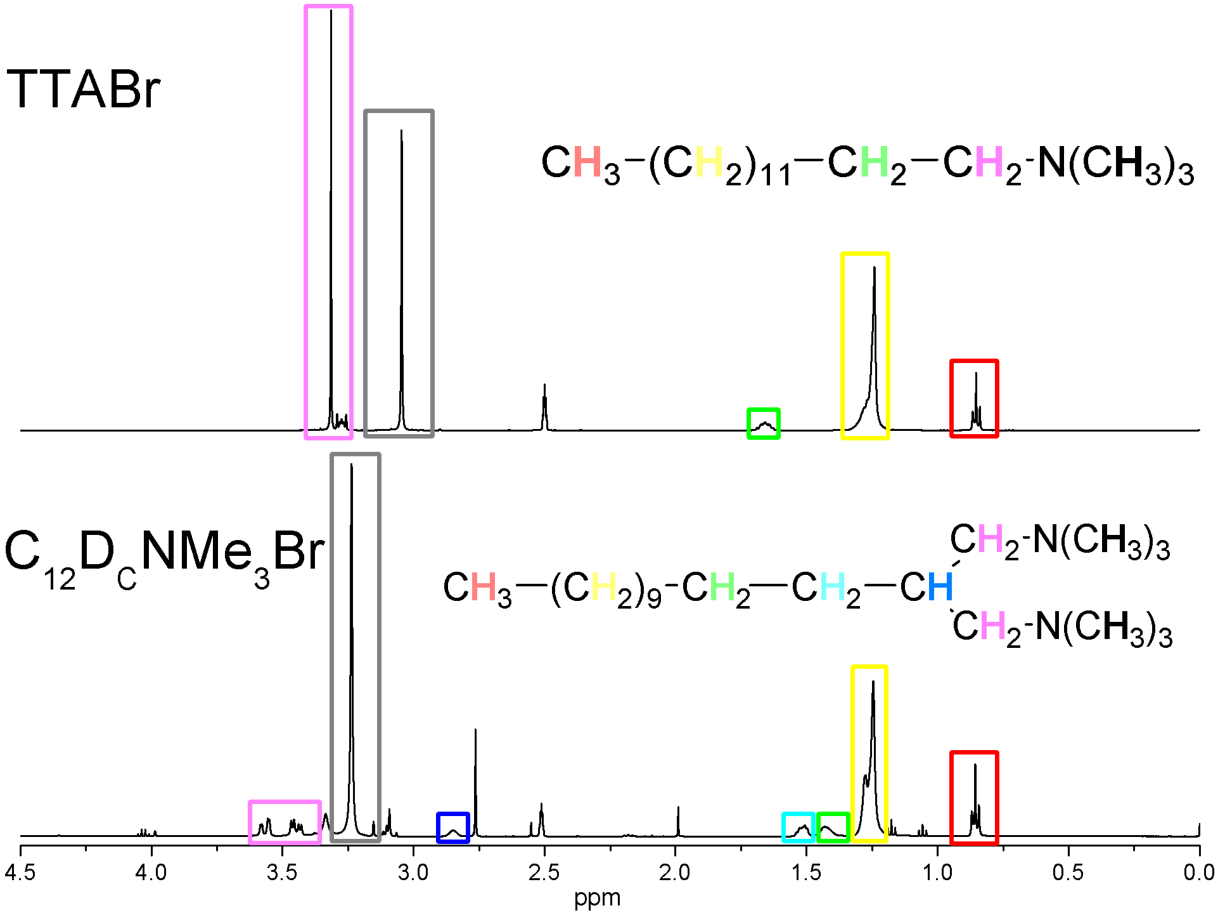

Figure S2. ^{1}H NMR spectra of parent non-magnetic surfactants: TTABr and $C_{12}D_CNMe_3Br$.

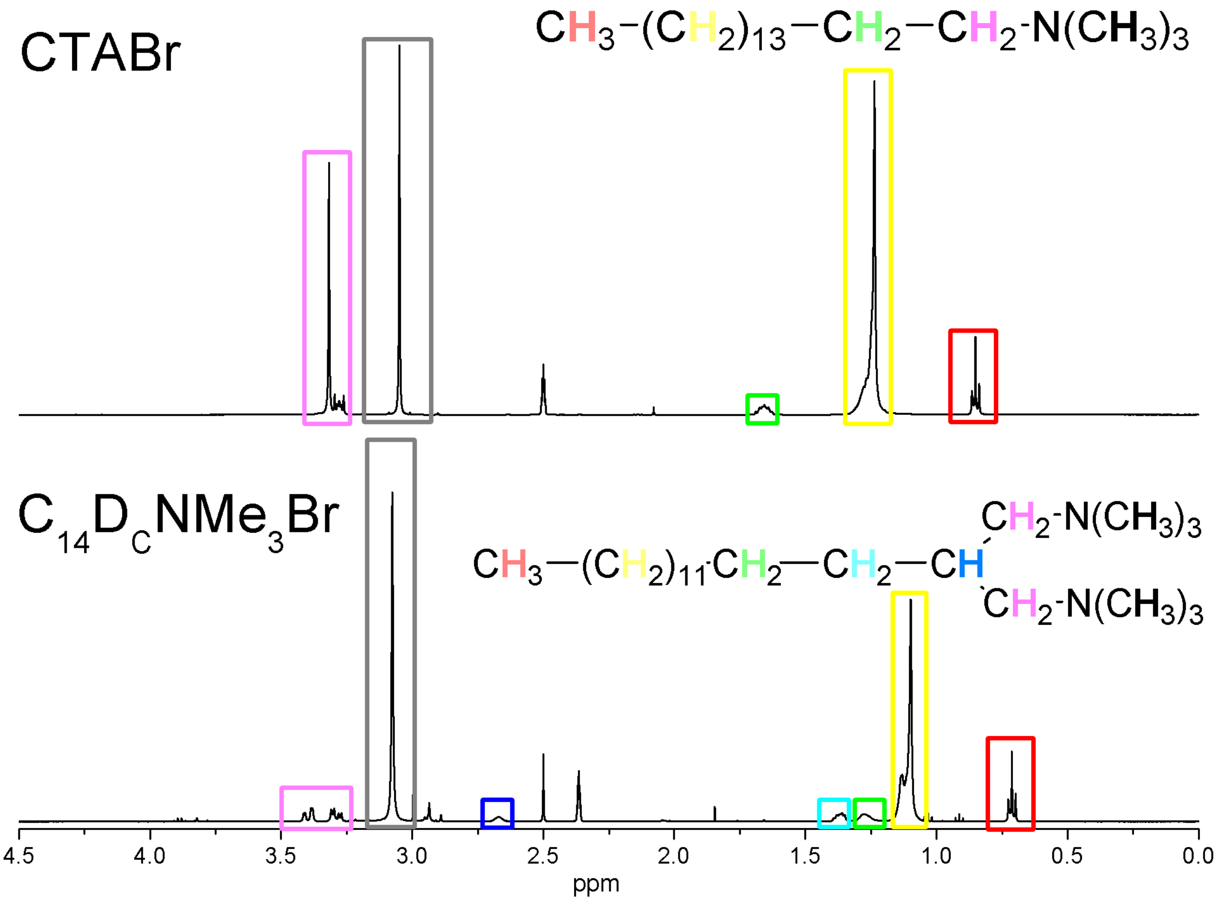

Figure S3. ^{1}H NMR spectra of parent non-magnetic surfactants: CTABr and $C_{14}D_CNMe_3Br$.

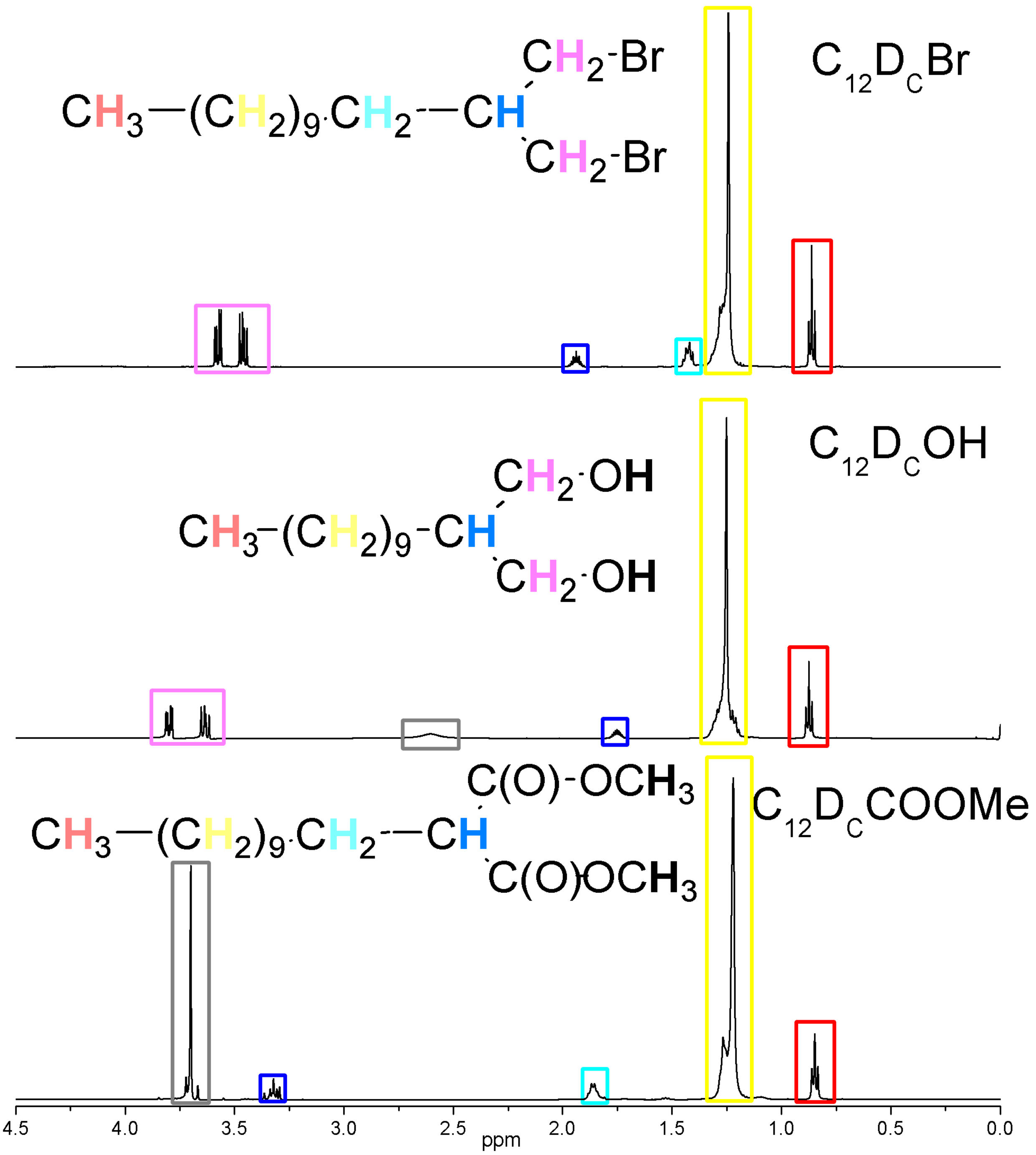

Figure S4. ^{1}H NMR spectra of particular intermediates, exemplified for the synthesis of $C_{12}D_CNMe_3Br$ – cf. Scheme 1.

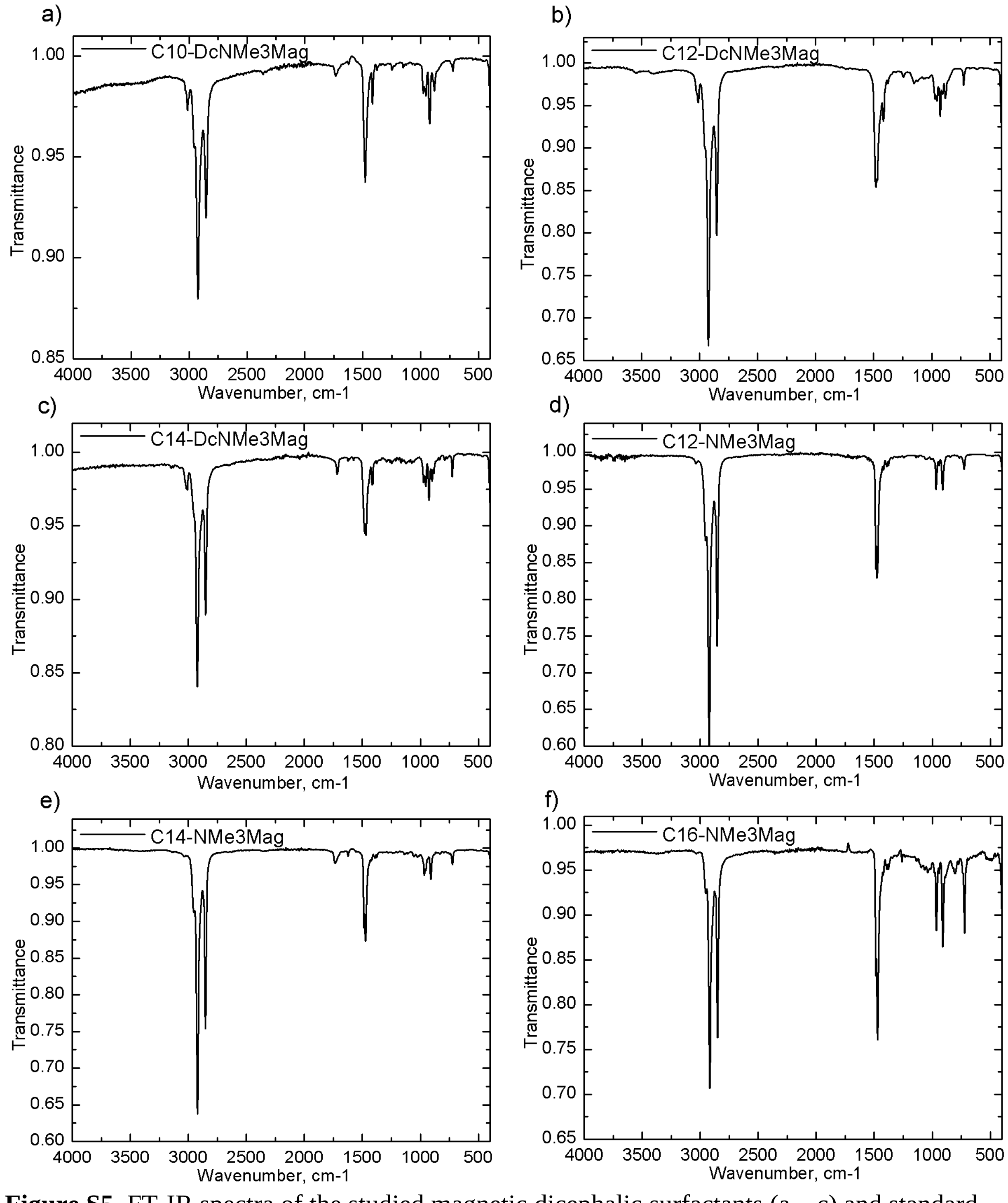

Figure S5. FT-IR spectra of the studied magnetic dicephalic surfactants (a – c) and standard magnetic ionic liquids surfactants (d – e).

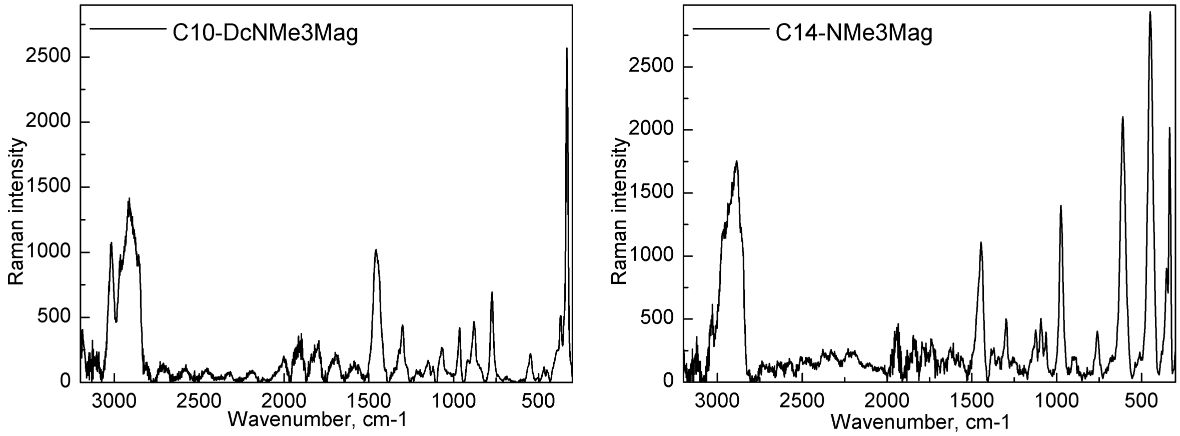

Figure S6. Examples of Raman spectra for C_{10}-$D_C$$NMe_3$Mag (left) and C_{14}-NMe_3Mag (right).

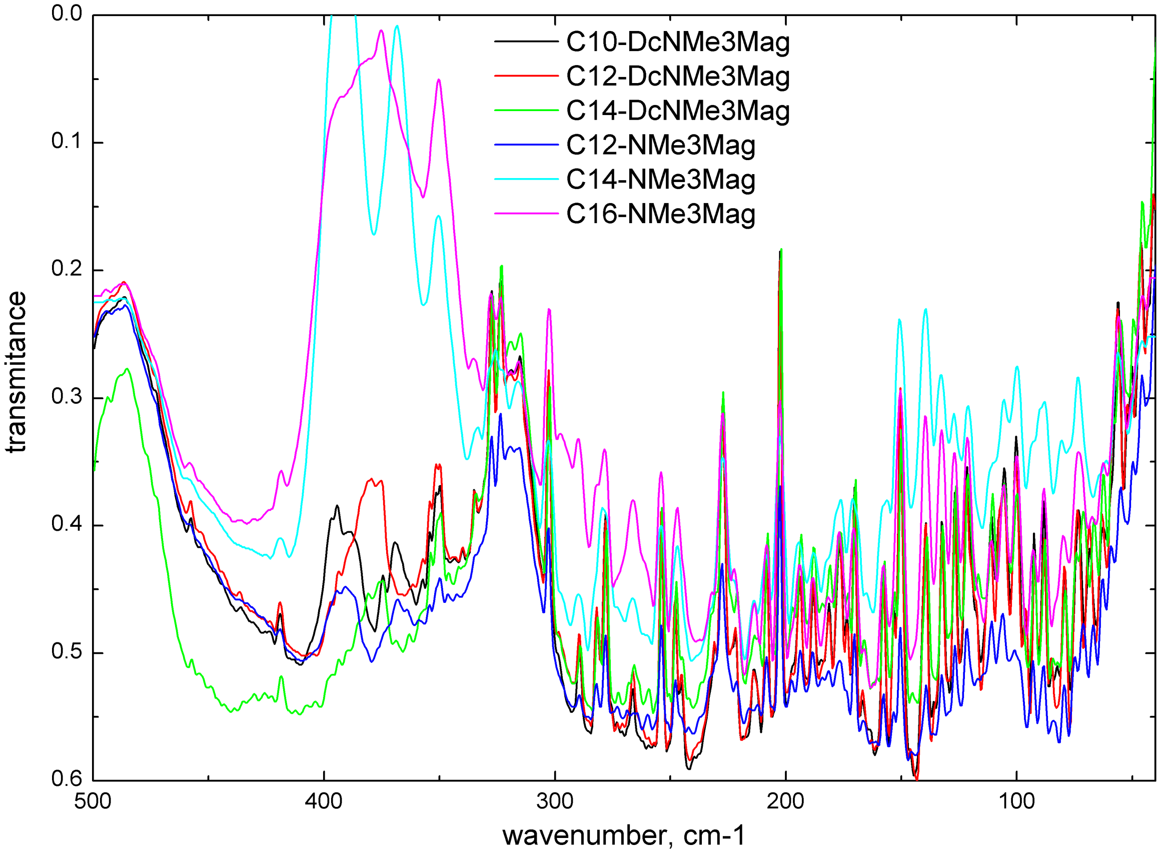

Figure S7. FIR spectra for the studied compounds.

4. TGA and DSC curves for $C_{10}D_CNMe_3Mag$, $C_{14}D_CNMe_3Mag$, $C_{12}NMe_3Mag$ and $C_{16}NMe_3Mag$

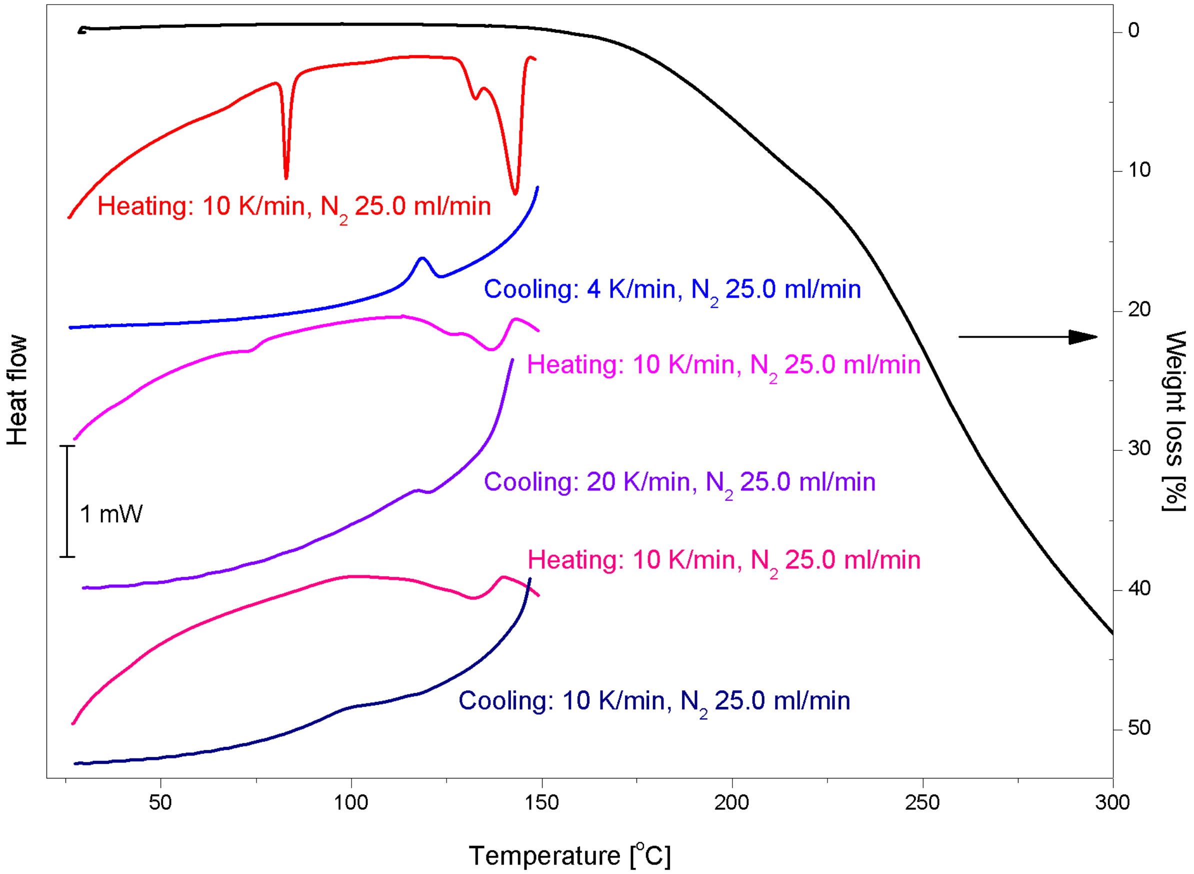

Figure S8. TGA (m = 7.3300 mg) and DSC (m = 2.7800 mg) curves for $C_{10}D_CNMe_3Mag$ – cf. Table 2.

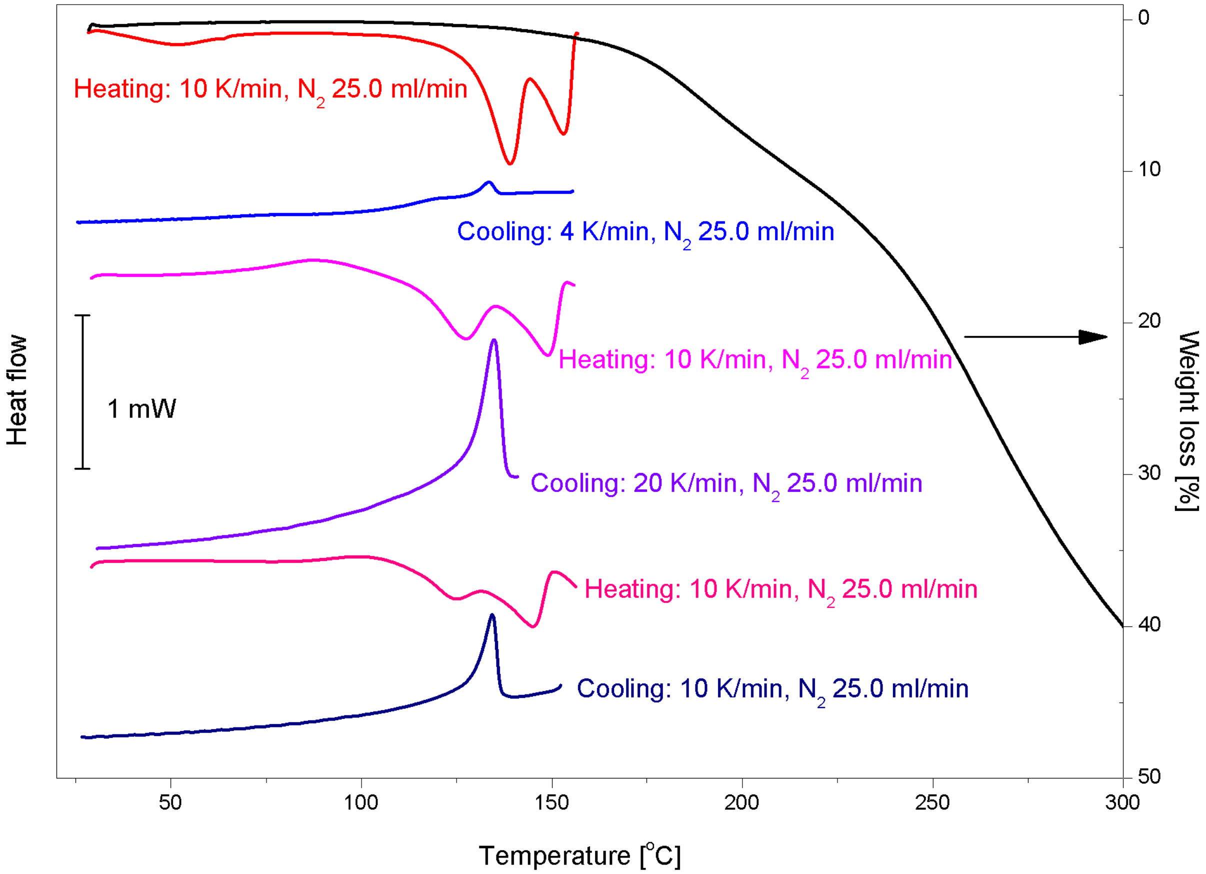

Figure S9. TGA (m = 6.7260 mg) and DSC (m = 2.2060 mg) curves for $C_{14}D_CNMe_3Mag$ – cf. Table 2.

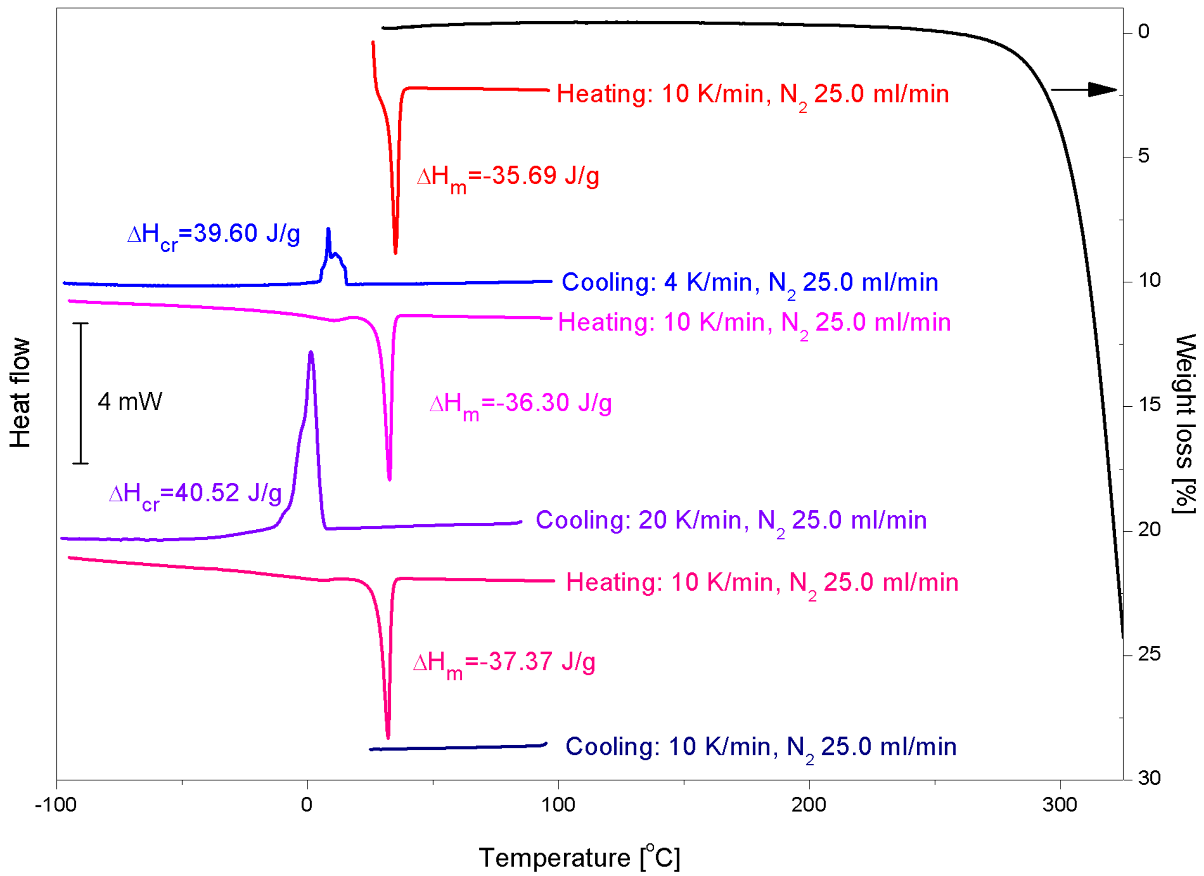

Figure S10. TGA (m = 9.4340 mg) and DSC (m = 2.8120 mg) curves for $C_{12}NMe_3Mag$ – cf. Table S1.

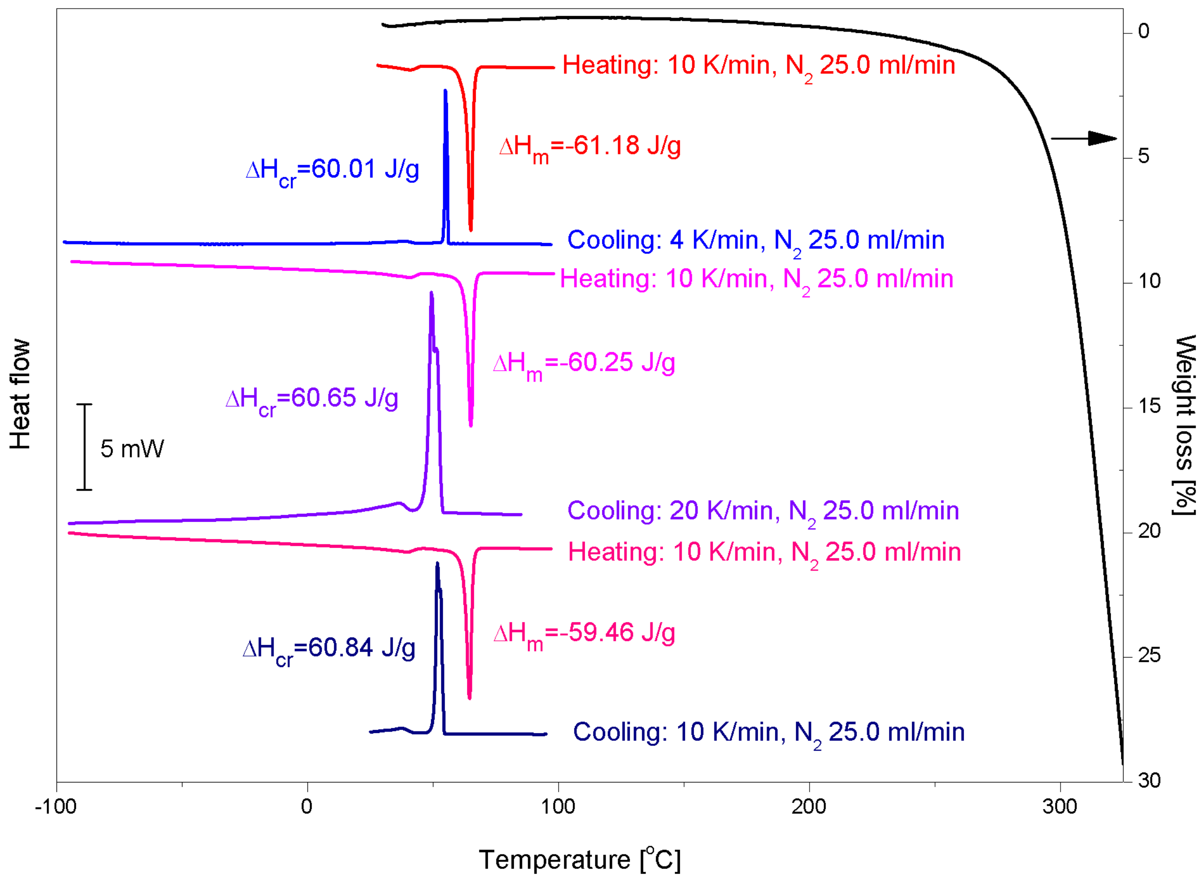

Figure S11. TGA (m = 7.3620 mg) and DSC (m = 2.4920 mg) curves for $C_{16}NMe_3Mag$ – cf. Table S1.

5. FT-IR spectra for C_{14}-NMe_3Mag (MILS) and C_{12}-$D_C$$NMe_3$Mag (Mag-D-Surf) before and after melting and solidification.

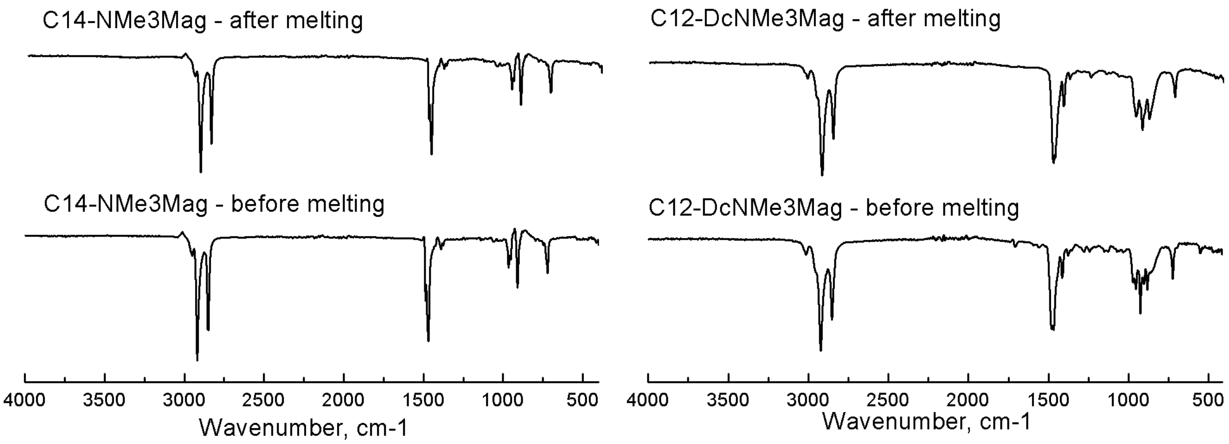

Figure S12. FT-IR spectra for C_{14}-NMe_3Mag (left) and C_{12}-$D_C$$NMe_3$Mag (right) before and after melting and solidification.
